# An allied reprogramming, selection, expansion and differentiation platform for creating hiPSC on microcarriers

**DOI:** 10.1111/cpr.13256

**Published:** 2022-05-19

**Authors:** Alan Tin Lun Lam, Valerie Ho, Svetlan Vassilev, Shaul Reuveny, Steve Kah Weng Oh

**Affiliations:** ^1^ Stem Cell Bioprocessing, Bioprocessing Technology Institute Agency for Science, Technology and Research Singapore Republic of Singapore

## Abstract

**Objectives:**

Induced pluripotent stem cells (iPSCs) generated by monolayer cultures is plagued by low efficiencies, high levels of manipulation and operator unpredictability. We have developed a platform, reprogramming, expansion, and differentiation on Microcarriers, to solve these challenges.

**Materials and Methods:**

Five sources of human somatic cells were reprogrammed, selected, expanded and differentiated in microcarriers suspension cultures.

**Results:**

Improvement of transduction efficiencies up to 2 times was observed. Accelerated reprogramming in microcarrier cultures was 7 days faster than monolayer, providing between 30 and 50‐fold more clones to choose from fibroblasts, peripheral blood mononuclear cells, T cells and CD34+ stem cells. This was observed to be due to an earlier induction of genes (β‐catenin, E‐cadherin and EpCAM) on day 4 versus monolayer cultures which occurred on days 14 or later. Following that, faster induction and earlier stabilization of pluripotency genes occurred during the maturation phase of reprogramming. Integrated expansion without trypsinization and efficient differentiation, without embryoid bodies formation, to the three germ‐layers, cardiomyocytes and haematopoietic stem cells were further demonstrated.

**Conclusions:**

Our method can solve the inherent problems of conventional monolayer cultures. It is highly efficient, cell dissociation free, can be operated with lower labor, and allows testing of differentiation efficiency without trypsinization and generation of embryoid bodies. It is also amenable to automation for processing more samples in a small footprint, alleviating many challenges of manual monolayer selection.

## Graphical abstract

We have developed an allied protocol for reprogramming, selecting, expanding and differentiating human pluripotent stem cells on Microcarriers (designated as RepMC). This method allows faster reprogramming, selecting 30‐50‐fold more candidates for characterization and also allows us to find high quality candidates that differentiate to cardiomyocytes and blood lineages. Mechanistically, this method appears to accelerate the induction, maturation and stabilization phases of reprogramming. Our findings help simplify the process of deriving and expanding iPSCs for therapeutic applications, offering a robust and scalable suspension platform for large‐scale generation of clinical grade iPSCs.

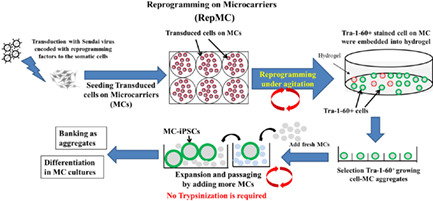

## 1 INTRODUCTION

Human‐induced pluripotent stem cells (iPSCs) are derived from adult somatic cells by the introduction of genes that encode pluripotent behaviour, defined by Takahashi and Yamanaka as Oct4, Sox2, Klf4 and c‐Myc (OSKM).^1^ Since then, several groups have reported iPSC reprogramming using different transduction methods to introduce the reprogramming factors into the cells, such as adenoviruses, lentiviruses, Sendai virus (SeV), mRNA/microRNA and episomal plasmids.^2^ SeV is a single‐stranded non‐integrative RNA virus which can replicate in the cytoplasm of infected cells. SeV‐mediated reprogramming is the most used integration‐free method of iPSC production available.^3^ It has been used for effective reprogramming of fibroblasts and peripheral blood mononuclear cells to iPSCs, with mean reprogramming efficiency of about 0.007%.^3^, ^4^, ^5^

Regardless of the approach, the manufacturing of iPSCs for therapeutic purposes relies on starting from somatic cell acquisition, cellular reprogramming, iPSC expansion, quality assurance, master/working cell banking and finally downstream directed differentiation to a relevant functional cell type. However, one major challenge is the production of high quality and adequate quantities of iPSCs for their applications. Conventional reprogramming approaches in static monolayer cultures have several disadvantages such as being labor‐intensive, time‐consuming for cell passaging and requiring cell dissociation to generate embryoid bodies (EBs) prior to differentiation.^1^, ^6^, ^7^ Importantly, the limited number of derived cells may be unable to support potential clinical applications.^8^ iPSC generation using conventional monolayer cultures typically takes 6–8 weeks, with varying degrees of efficiency depending on the method of reprogramming.^5^, ^9^, ^10^ In order to increase the reprogramming efficiency and ultimately scale up the production of these cells, researchers have tried to use bioreactor suspension culture to induce pluripotency of mouse fibroblasts to mouse iPSCs in the form of cell aggregates.^11^, ^12^, ^13^ However, it is not yet clear whether the suspension culture approach will work well for human cells.

Our group has demonstrated significant progress in the study of microcarrier (MC) cultures for iPSC expansion and differentiation.^14^, ^15^ MC cultures are favourable for maintaining stem cell proliferation without spontaneous differentiation after 10 passages^14^, ^16^, ^17^ and are characterized by a high surface‐to‐volume ratio which allows for high density cell culture.^18^ Utilizing the full potential of MC cultures could help simplify the process of deriving and expanding iPSCs for therapeutic applications, offering a robust and scalable suspension platform for large‐scale generation of clinical grade iPSCs.

Here, we examined whether MC cultures provide a selective advantage to enhance iPSC reprogramming and selected for iPSC with efficient differentiation abilities. We demonstrate that suspension MC cultures with agitation significantly improved the reprogramming efficiency for both adherent and suspension human somatic cells. The resulting MC‐iPSCs possess pluripotency and robust differentiation characteristics and display a normal karyotype. By applying this approach to somatic fibroblasts, as well as peripheral blood mononuclear cells (PBMC), CD3+ T‐cells, and CD34+ haematopoietic progenitor cells, hundreds of fully reprogrammed iPSCs can be derived, providing ~50‐fold more clones/candidate iPSCs than conventional adherent culture methods. The resulting microcarrier‐derived iPSCs (MC‐iPSCs) resemble embryonic stem cells in their in vitro characteristics, including gene expression and differentiation potential. We believe this MC reprogramming approach has the added potential to enhance other areas of iPSC research such as CRISPR edited clone selection.

Previous studies have also reported that the increased tumorigenicity of certain iPSC lines in both chimeric mice and their germline‐transmitted progeny can be observed as a result of c‐Myc reactivation.^19^, ^20^, ^21^ Although c‐Myc can be omitted, conventional reprogramming efficiency drops approximately 100‐fold.^21^ To this end, we also investigated if we could still derive iPSC with c‐Myc elimination at high efficiency on the MC platform.

## 2 MATERIALS AND METHODS

For further details of this section, please refer to the Supplemental Experimental Procedures.

### 2.1 Sendai virus (SeV) reprogramming

**FIGURE 1:**
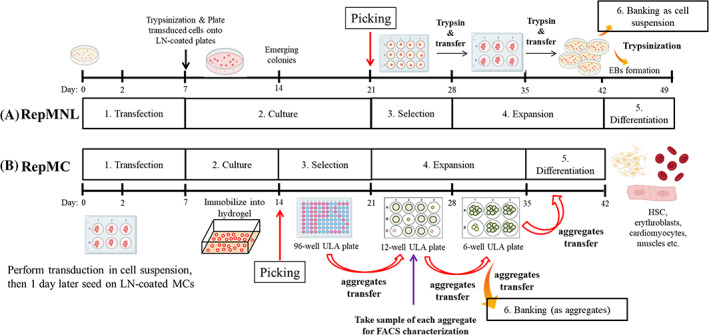
A schematic diagram for somatic reprogramming in (A) *RepMNL* and (B) *RepMC* approaches

Figure 1 visual representation of reprogramming methods.

#### 2.1.1 Reprogramming by conventional method in monolayer cultures (*RepMNL*; Figure 1A)

Adherent HFF‐01 and IMR90 fibroblasts, as well as suspension PBMC (all from ATCC), CD3+ T‐cells and CD34+ cells (isolated from PBMC, please refer to Supplemental Experimental Procedures) were transduced with CytoTune®‐iPS 2.0 Sendai virus (SeV) Reprogramming kit (ThermoFisher Scientific), using a MOI 5:5:3 (hKOS:hc‐Myc:hKlf4) and following the manufacturer recommendations.

#### 2.1.2 Reprogramming by novel approach in microcarrier cultures (*RepMC*; Figure 1B)

Single‐cell suspensions of 3 × 10^5^ HFF‐1, IMR90, PBMC, CD3+ T‐cells or CD34+ cells were plated per well of a 6‐well Ultra‐low attachment (ULA) plate with the corresponding cell growth medium. Subsequently, the cell suspension was transduced with CytoTune®‐iPS 2.0 SeV Reprogramming kit, using a MOI 5:5:3 (hKOS:hc‐Myc:hKlf4) and placed in a 37°C, 5% CO_2_ shaker incubator (New Brunswick™ S41i Incubator Shaker) under agitation (100–110 rpm) (day 0). After 24 h, the transduced cells were transferred to a well of 6‐well ULA containing 20 mg of Laminin521 (LN; Biolamina)‐coated MCs and Essential 8™ medium (E8; ThermoFisher Scientific). The cell‐covered‐MC (cell‐MC) were cultured in the CO_2_ shaker incubator under agitation (100–110 rpm) with E8 medium changed every other day for a week. Following, the cell‐MC were collected, resuspended in mTeSR™1 medium (mT; StemCell Technologies), and mixed with thermoreversible (TGP; Mebiol Inc) hydrogel on ice. Immediately, the cell‐hydrogel mixture was transferred evenly into 6 wells of a 6‐well tissue culture plate and incubated in a 37°C, 5% CO_2_ incubator for 7 days with daily mT medium changes.

#### 2.1.3 Live‐cell immunofluorescence staining of TRA‐1‐60 positive cells on MC in hydrogel

Live‐staining with StainAlive TRA‐1‐60 (DyLight™488; Stemgent) was used to identify the onset of TRA‐1‐60 expression, a marker associated with pluripotency. The TRA‐1‐60‐stained cells were identified using ClonePix™ System (Molecular Devices).

#### 2.1.4 Selection and expansion of TRA‐1‐60 positive cell‐MC

The marked cell‐MC were picked and transferred into a separate well of a 96‐well ULA plate (with 200 μl mT and 0.5 mg LN‐coated MCs). The 96‐well ULA plate was then incubated in a 37°C 5% CO_2_ incubator for 7 days under static conditions. On day 7, live‐staining with StainAlive TRA‐1‐60 was performed again in the 96‐well ULA plate to identify the growing pluripotent cells on MCs (size increase at least 2× that of the initial aggregate) under a fluorescence microscope, which were then transferred into a separate well of a 12‐well ULA plate (with 3 ml mT medium and 8 mg LN‐coated MCs). The 12‐well ULA plate was then incubated in a 37°C 5% CO_2_ incubator for another 7 days under static conditions. After 7‐days incubation, fast‐growing cell‐MC aggregates (size increase at least 2× of initial aggregate plated in the 12‐well ULA plate) were selected and transferred into a separate well of 6‐well ULA plate (with 5 ml mT medium and 20 mg LN‐coated MCs). The cell aggregates should break down into smaller aggregates gently by the 1 ml pipette tips. The 6‐well ULA plate with cell‐MC aggregates was then incubated in a 37°C 5% CO_2_ incubator for another 7 days under agitation (100–110 rpm). The expanded cell‐MC aggregates (MC‐iPSCs) were then harvested for characterization.

## 3 RESULTS

### 3.1 iPSC generation by conventional method in MNL cultures (*RepMNL*)

Adherent fibroblasts HFF‐01, IMR90, suspended haematopoietic cells (PBMC), CD3+ T‐cells and CD34+ cells were transduced with SeV reprogramming factors using the conventional MNL method (designated as *RepMNL*) following the manufacturer's instructions, except for LN being used as an adhesive substrate rather than vitronectin. iPSC‐like colonies (designated as MNL‐iPSCs) began appearing at day 12 post‐transduction. Table 1 shows the transduction efficiencies and reprogramming efficiencies of the cells, respectively, as calculated by the number of TRA‐1‐60+ colonies emerging on day 14 per initial cell seeding.

**TABLE 1:** Comparison between *RepMNL* and *RepMC*

	Transduction efficiency (%)		Reprogramming efficiency (%)		Total no. of TRA‐1‐60+ cells	
	*RepMNL*	*RepMC*	*RepMNL*	*RepMC*	*RepMNL*	*RepMC*
Adherent fibroblasts						
HFF‐01	33.4 ± 6.3	68.4 ± 4.6**	0.04 ± 0.02	0.97 ± 0.01****	40 ± 4	1983 ± 179****
IMR90	33.7 ± 1.3	65.5 ± 1.7***	0.03 ± 0.01	0.61 ± 0.04***	34 ± 1	1203 ± 151***
3F‐HFF01	36.9 ± 1.9	69.4 ± 6**	0.0009 ± 0.004	0.15 ± 0.03**	3 ± 1	463 ± 97***
Fold increase: *RepMC* vs. *RepMNL*						
HFF‐01		2.1 ± 0.4		24.5 ± 5.9		50.1 ± 9.1
IMR90		1.9 ± 0.1		18.0 ± 2.2		34.9 ± 3.4
3F‐HFF01		1.9 ± 0.2		184.7 ± 43.5		184.7 ± 43.5
Suspended haematopoietic cells						
PBMC	59.1 ± 5.9	60.7 ± 1.4	0.02 ± 0.01	0.97 ± 0.1****	36 ± 2	1757 ± 185****
CD3+ T‐cells	48.5 ± 5.5	60.5 ± 1.4	0.02 ± 0.001	0.85 ± 0.05****	30 ± 7	1544 ± 131****
CD34+ cells	46.9 ± 2.9	52.9 ± 2.6	0.02 ± 0.01	0.5 ± 0.1***	20 ± 8	787 ± 95***
3F‐PBMC	57.0 ± 2.8	59.8 ± 0.8	0.0006 ± 0.0002	0.11 ± 0.01****	2 ± 1	418 ± 16****
Fold increase: *RepMC* vs *RepMNL*						
PBMC		1.0 ± 0.1		47.6 ± 7.5		49.0 ± 7.0
CD3+ T‐cells		1.3 ± 0.2		41.3 ± 3.7		52.1 ± 7.8
CD34+ cells		1.1 ± 0.1		36.4 ± 9.2		41.2 ± 10.5
3F‐PBMC		1.1 ± 0.04		187 ± 59.9		186.7 ± 46.3

	Duration of the process (6 passage)	No. of trypsinization (6 passage)	Formation of EBs
Other process benefits			
*RepMNL*	~10 weeks	~6 times	Need trypsinization
*RepMC*	~8 weeks	No need (passage as aggregates)	No trypsinization is necessary, iPSC differentiation can be done directly in MC suspension culture

*Note*: Transduction efficiency, reprogramming efficiency and total number of TRA‐1‐60+ clones emerged at day 14 were shown. Mean ± SD. (*n* = 3), with **p < 0.01, ***p < 0.001, and ****p < 0.0001 compared to *RepMNL*.

Four MNL‐iPSC colonies randomly isolated (Figure S1A), were passaged at least 6 times by trypsinization in order to obtain sufficient cells for analysis. The cells were analysed for expression of Oct4, TRA‐1‐60, and SSEA‐4 (Figure S1B), RT‐qPCR analysis of expression of differentiation‐associated genes (Figure S1C), spontaneous differentiation (Figure S1D) and karyotype (Figure S1E). Results demonstrated that all lines tested exhibited pluripotency and could differentiate into the three germ layers. However, it is worth mentioning that MNL01 showed an abnormal trisomy in chromosome 12, whereas MNL02 to MNL04 had a normal diploid karyotype.

Monolayer reprogramming is an inefficient process, taking an average of 8–10 weeks to generate sufficient cells for characterization and banking, with a limited number of colonies established. Karyotype abnormalities can sometimes also be observed (Figure S1E).^22^ Moreover, technical limitations abound, such as time‐consuming passaging by enzymatic cell dissociation and the difficulty of testing the differentiation efficiency in EB culture in the mid‐phase of cell line development. To overcome these challenges, we set out to design a more efficient reprogramming platform using MCs, named *RepMC*.

### 3.2 Reprogramming by *RepMC* approach enhances transduction and reprogramming efficiencies

First, a non‐agitated *RepMC* approach was performed on HFF‐01 fibroblasts. However, the reprogramming efficiency was much lower than that observed in agitated cultures (Table S1). Therefore, we chose to move forward with the agitated *RepMC* method for further study.

Table 1 shows higher transduction efficiency of HFF‐01 and IMR90 in agitated *RepMC* when compared to *RepMNL* (*p* < 0.01). Most importantly, the efficiency of reprogramming using *RepMC* was ~20‐fold higher over *RepMNL* (*p* < 0.01). Moreover, about 1000–2000 TRA‐1‐60+ colonies were obtained by day 14 with *RepMC*, compared to only about 35–40 colonies obtained using *RepMNL*. In short, we achieved higher iPSC generation efficiency using the *RepMC* approach as compared to *RepMNL*.

We next attempted to reprogram suspended haematopoietic cells (PBMC, CD3+ T‐cells, and CD34+ cells) into iPSCs. Table 1 shows that *RepMC* exhibits higher reprogramming efficiencies when compared with *RepMNL* (*p* < 0.0001), with reprogramming efficiency of *RepMC* for suspension blood cells being ~40‐fold higher than *RepMNL*. However, it is worth noting that there were no significant differences in transduction efficiencies between *RepMC* and *RepMNL* (*p* > 0.02) for haematopoietic cells.

**FIGURE 2:**
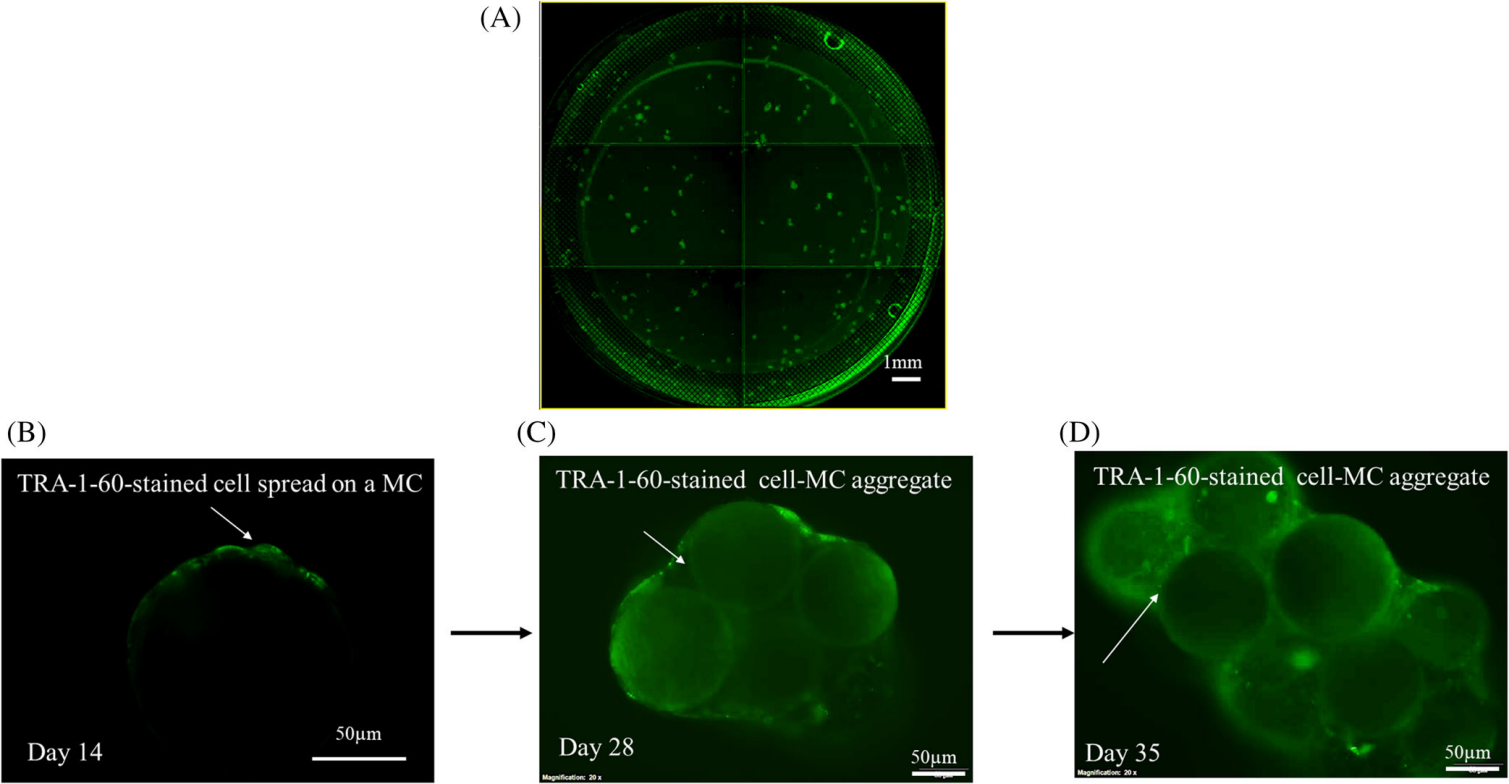
Generation of HFF‐01‐derived MC‐iPSCs with *RepMC* method. (A) A representative microscopic view of a well of a 6‐well plate of cell‐MC. Green colour dots illustrated the TRA‐1‐60+ cell‐MC. Scale bar = 1 mm. (B) An individual TRA‐1‐60‐stained cell‐covered MC in TGP hydrogel at day 14. Scale bar = 50 μm. White arrow indicates the expanded colony of cells from a single cell spread on a single MC. (C) A representative image of cell‐covered‐MC aggregate expanded in 12‐well ULA plate at day 28. Scale bar = 50 μm. White arrows indicate cell growth between MCs. (D) A representative image of cell‐covered‐MC aggregate expanded in 6‐well ULA plate at day 35. Scale bar = 50 μm. White arrows indicate cell growth between MCs

**FIGURE 3:**
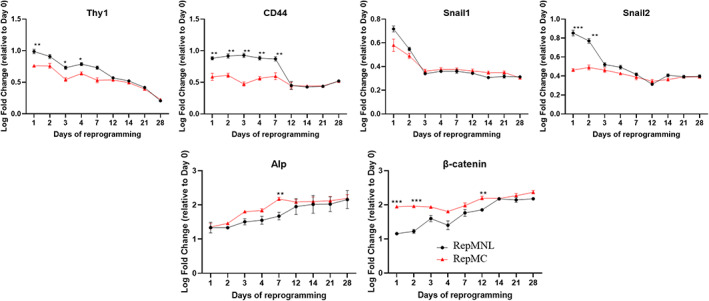
Gene expression profiles during the initiation phase of HFF‐01 fibroblast reprogramming by qPCR. Log fold‐changes relative to day 0 fibroblasts are depicted for *RepMNL* and *RepMC* cultures. Expression levels that differ significantly at matching timepoints were depicted by horizontal brackets (*T*‐test *p < 0.01; **p < 0.001; ***p < 0.0001). Error bars SD (*n* = 3)

**FIGURE 4:**
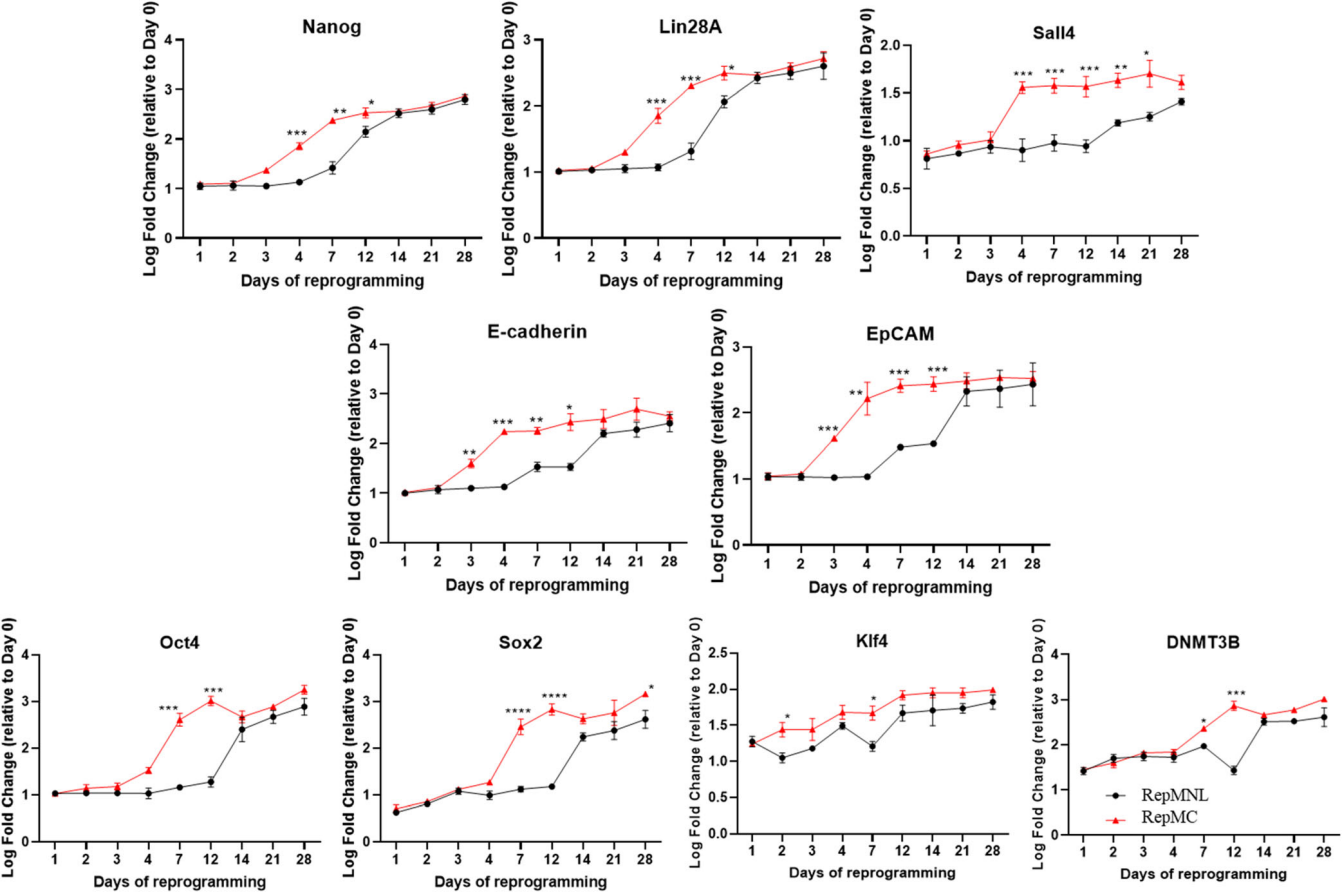
Gene expression profiles during the maturation and stabilization phases of HFF‐01 fibroblast reprogramming determined by qPCR. Log fold‐changes relative to day 0 fibroblasts are depicted for both *RepMNL* and *RepMC* cultures. Expression levels that differ significantly at matching timepoints were depicted by horizontal brackets (*T*‐test *p < 0.01; **p < 0.001; ***p < 0.0001). Error bars SD (*n* = 3)

**FIGURE 5:**
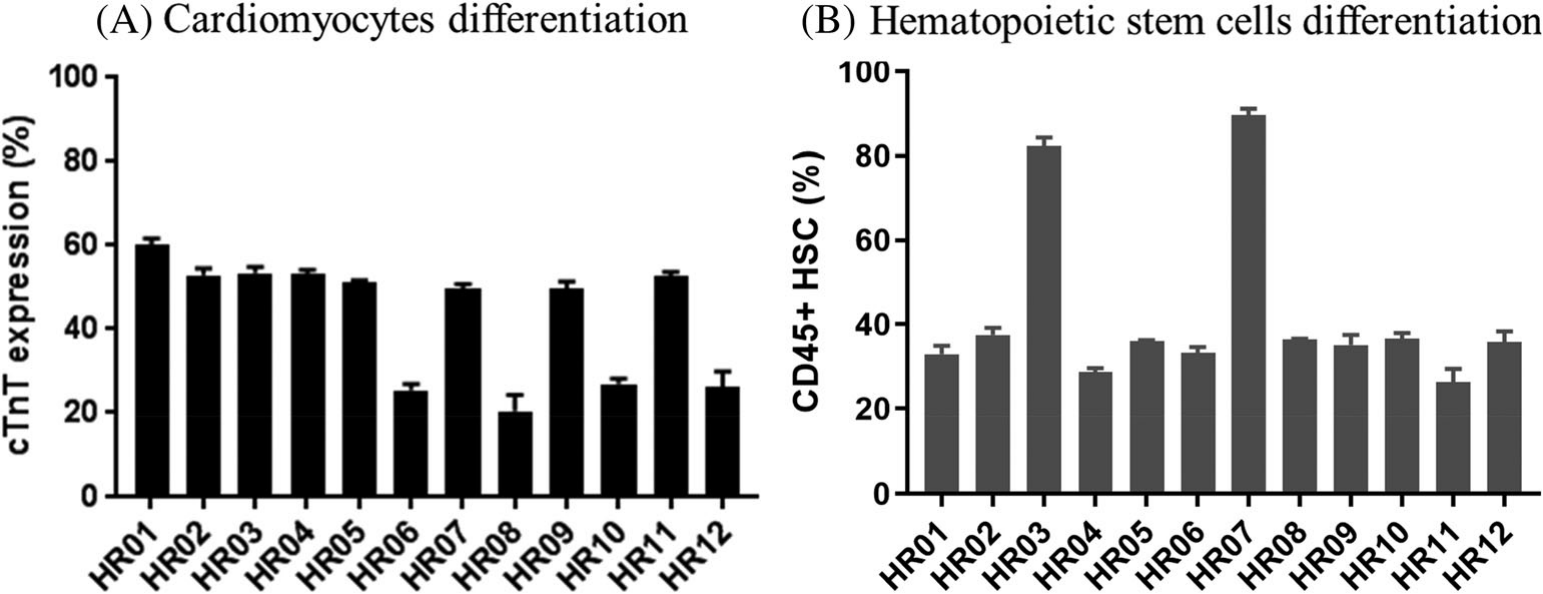
Directed differentiation of HFF‐01‐derived MC‐iPSCs (HR01 to HR12) towards cardiomyocytes and erythroblasts, in *RepMC*. (A) Cardiac specific marker (cardiac troponin‐T; cTnT) was measured using flow cytometry at day 14 of the Wnt modulation‐based cardiomyocyte differentiation. Four clones (HR06, HR08, HR10 and HR12) exhibited <20% cTnT, while other clones expressed ~60% cTnT. (B) CD45+ cells were monitored by flow cytometry at day 11 of the BMP4‐based erythroblast differentiation. HR03 and HR07 expressing >80% CD45+, whereas other clones expressed <40%

**FIGURE 6:**
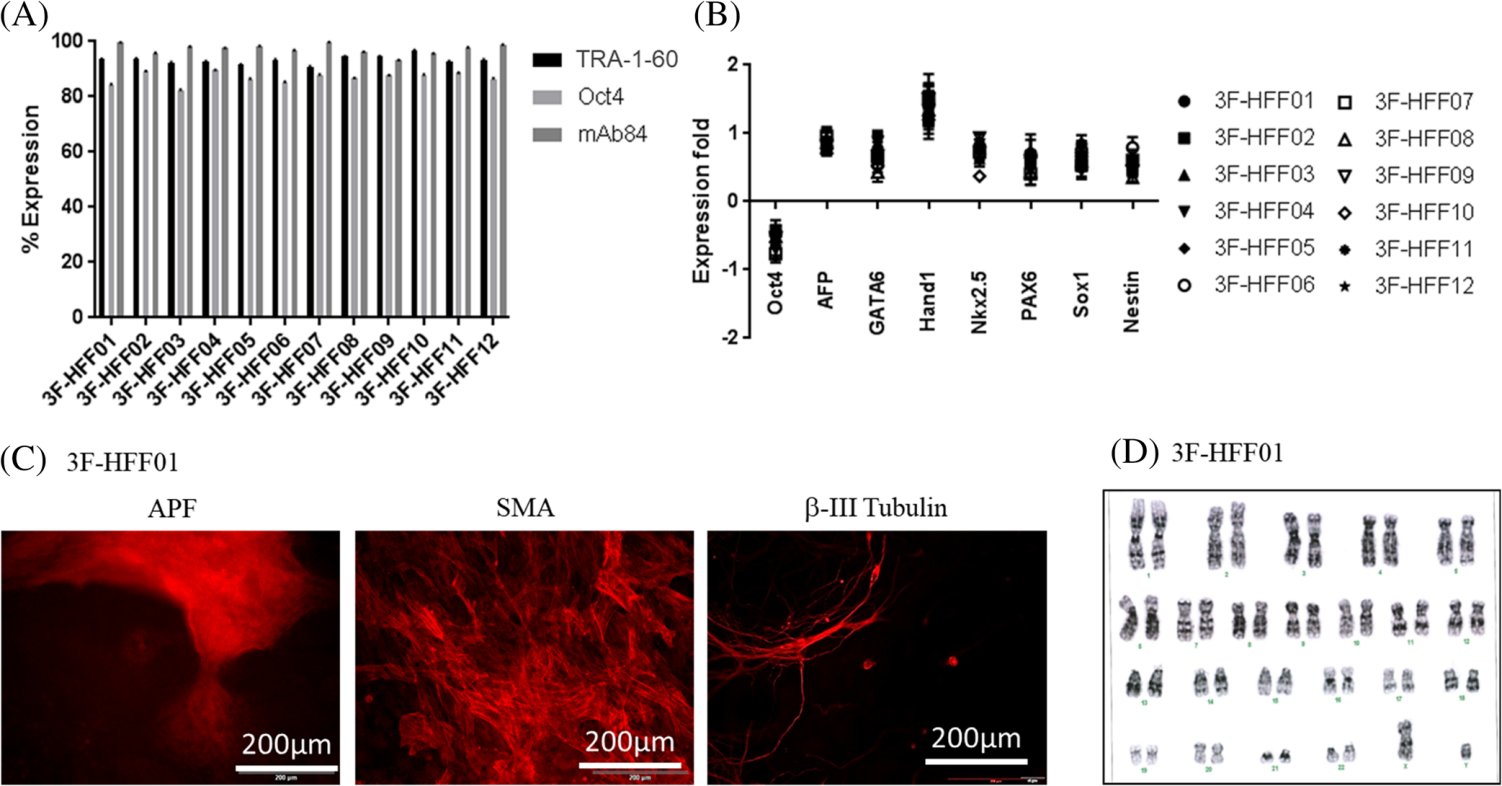
Reprogramming of HFF‐01 fibroblast by 3‐factors only (Oct4, Sox2 and Klf4) on *RepMNL* and *RepMC* cultures. (A) Flow cytometry analysis showing expression of pluripotent markers (TRA‐1‐60, Oct4 and SSEA‐4) in the reprogrammed MC‐iPSCs (3F‐HFF01 to 3F‐HFF12). (B) Log fold‐changes of pluripotent and three germ‐layer‐specific genes compared with undifferentiated MNL‐iPSCs. (C) Staining of in vitro differentiated MC‐iPSCs (3F‐HFF01) for markers of mesoderm (SMA, α‐smooth muscle Actin), ectoderm (β‐III tubulin) and endoderm (AFP, α‐fetoprotein). Scale bars: 200 μm. (D) Karyotyping of a representative clone (3F‐HFF01). Normal 46 XY karyotypes by G‐banding, 20 metaphase spreads were counted per sample

Figure 2A shows an example of the microscopic view of a well of a 6‐well plate with immobilized HFF‐01 cell‐MC in the TGP hydrogel taken in the ClonePix™ System at day 14, with green colour dots showing the live TRA‐1‐60+ cell attached and spread on MC. Single TRA‐1‐60+ cell‐MC embedded in the TGP hydrogel (Figure 2B) were then randomly picked by the ClonePix™ System and transferred to a separate well of a 96 ULA plate containing 0.5 mg LN‐coated MCs. Thereafter, subsequent cell expansion and passaging, from 96‐well ULA to 12‐well ULA (Figure 2C) to 6‐well ULA (Figure 2D), were done by simply picking and transferring a fraction of cell‐MC aggregates to new LN‐coated MCs, without the need for trypsinization. The expanded TRA‐1‐60+ cell‐aggregates in 6‐well ULA culture (Figure 2D) were designated as MC‐iPSCs. This demonstrates that the use of cell dissociation solutions or cell scraping is not required for passaging in the *RepMC* approach. Moreover, differentiation of the cell‐MC aggregates was performed by simply sampling a fraction of aggregates and transferring them to differentiation medium without the 3 additional steps of replating, cell dissociation and EB formation.

### 3.3 *RepMC* promotes iPSC generation by facilitating gene activation early in reprogramming

To understand the effect of agitated *RepMC* culture on reprogramming progression, we performed RT‐qPCR studies on genes commonly expressed during the phases of reprogramming^23^, ^24^ with cells harvested at discrete stages of reprogramming (days 1, 2, 3, 4, 5, 7, 14, 21, and 28) from the *RepMNL* and *RepMC* of HFF‐01 (Figures 3 and 4). Our results for all reprogramming approaches correlate well with the literature reported sequential molecular events in somatic cells^23^, ^24^: (1) Initiation phase: downregulation of the fibroblast‐specific surface markers (such as *Thy1* and *CD44*, Figure 3), coupled with a loss of mesenchymal cell signature (such as *Snail1/2* Figure 3), and particularly induction of the signal transducer *β‐catenin* and *Alkaline Phosphatase* (Alp) (Figure 3); (2) Maturation phase: upregulation of endogenous *Nanog* and *Lin28*, Wnt effector *Sall4*, epithelial genes *EpCAM*, and *E‐cadherin* (Figure 4); and, finally, (3) Stabilization phase: acquisition of full pluripotency signature such as expression of endogenous *Oct4* and *Sox2*, *Klf4* and DNA methyltransferase 3B (*DNMT3B*) (Figure 4).

We found that the *RepMC* culture accelerated the early expression of *β‐catenin* on day 1, at least 2 days earlier than was observed in *RepMNL* (Figure 3). Early and high induction of *E‐cadherin* and *EpCAM* on day 3 was also observed, whereas *RepMNL* only showed induction on day 7 (Figure 4). *Nanog*, *Lin28A* and *Sall4* were also expressed as early as day 4, compared to *RepMNL* which only reached equivalent levels at day 12 or later (Figure 4). Endogenous *Oct4* and *Sox2* were induced on day 7, earlier than in *RepMNL*, which showed expression on day 14. Epigenetic gene *DNMT3B* was also expressed at least 2 days earlier (Figure 4). It is also interesting to note that Klf4 was already induced on day 2 in *RepMC*, compared to *RepMNL* which only reached similar levels at day 4 (Figure 4). These data suggest that *RepMC* accelerated iPSC generation through an earlier induction of the epithelial–mesenchymal transition (EMT) and mesenchymal–epithelial transition (MET) processes.

To further confirm the changes in gene expression over time during initialization (days 1, 3, 4, and 7), we performed the same analysis of the expression of reprogramming genes in PBMC (Figure S2), which has similar transduction efficiency using *RepMNL* or *RepMC*. Similar to HFF‐01, early expression of *β‐catenin* on day 1, followed by high induction of *E‐cadherin* and *EpCAM* on day 3, and subsequent expression of *Nanog*, *Lin28A*, *Sall4*, and *Klf4* at day 4 were observed in PBMC reprogrammed by *RepMC* (Figure S2).

### 3.4 Characterization of MC‐iPSCs

Following *RepMC* reprogramming, 60 MC‐iPSCs from 5 somatic origins: HFF‐01, IMR90, PBMC, CD3+ T‐cells and CD34+ cells (12 clones from each source) were further characterized. The cells were analysed for expression of Oct4, TRA‐1‐60, and SSEA‐4 (Figure S3), RT‐qPCR analysis of expression of differentiation‐associated genes (Figure S4), spontaneous differentiation by immunostaining (Figure S5) and karyotyping (Figure S6). It is worth to note that all 10 karyotypes were normal. Testing revealed that Sendai virus was retained in MC‐iPSCs for up to 10 passages and was completely absent in p15 cells, with similar findings observed in MNL‐iPSCs (Figure S7). Results show that all lines tested exhibited pluripotency, had the capacity to differentiate into the three germ layers and had a diploid karyotype. It is noteworthy that variations in gene expression levels between different samples and cell sources were observed during analysis of differentiation‐associated genes.

To further confirm the development potential of the MC‐iPSCs, a fraction of 12 HFF‐01‐derived MC‐iPSCs suspension cultures was collected for cardiomyocyte differentiation using our previously published protocol.^25^, ^26^ Although all differentiated HFF‐01‐derived MC‐iPSCs examined were positive for expression of the cardiomyocyte marker cTnT (Figure 5A), 4 clones (HR06, HR08, HR10 and HR12) exhibited lower levels of cTnT (<20%) than the other 8 clones (~60%).

We also compared the erythroblast differentiation potential of the same 12 HFF‐01‐derived iPSCs following our published blood differentiation protocols.^27^, ^28^ Derived erythroblast clones were functional and had oxygen carrying capacity (data not shown). Although all could differentiate to haematopoietic cells and subsequently erythroblasts, the haematopoietic potential varied between clones with 2 expressing CD45 (a marker of haematopoiesis) to a high degree (>80%; Figure 5B). Importantly, clones 3 and 7 could be selected for efficient differentiation towards both cardiomyocytes and blood lineage cells (Figure 5A,B).

In summary, we have demonstrated that *RepMC* shows higher reprogramming efficiency compared to *RepMNL* with 5 cell sources (adherent and suspension). We confirmed MC‐iPSC clones exhibited high levels of pluripotency and maintained their differentiation potential for all three germ layers as well as robust differentiation to cardiomyocytes and blood lineages.

### 3.5 Expedited derivation of 3F‐MC‐iPSCs (OKS) in *RepMC* cultures

Previous studies have reported that the increased tumorigenicity of certain iPSC lines in both chimeric mice and their germline‐transmitted progeny is the result of c‐Myc reactivation.^19^, ^29^ Thus, we tested *RepMC* reprogramming with three factors: Oct4, Sox2 and Klf4 (c‐Myc being eliminated and only hKOS and hKlf with MOI 5:3 were added) using *RepMNL* as control, in HFF‐01 and PBMC. In *RepMNL*, while reprogramming efficiency was 0.02%–0.04% with 4 factors and efficiency was 0.0006%–0.0009% with 3 factors (Table 1). This is in agreement with previous publications showing that c‐Myc exclusion normally leads to an approximately 100‐fold drop in conventional monolayer reprogramming efficiency.^21^ Notably, reprogramming efficiency with 3 factors was about 185 and 187‐fold higher in *RepMC* than in *RepMNL*, in 3F‐HFF‐01 and 3F‐PBMC, respectively (Table 1), resulting in more positive clones emerging at day 14 in *RepMC* vs *RepMNL*.

Twelve clones obtained by 3 factors transduction of HFF‐01 were analysed for expression of Oct4, TRA‐1‐60 and SSEA‐4 (Figure 6A), RT‐qPCR analysis of expression of differentiation‐associated genes (Figure 6B), spontaneous differentiation (Figure 6C), and karyotyping (Figure 6D). All lines tested were pluripotent, had the capacity to differentiate into the three germ layers and had a diploid karyotype.

## 4 DISCUSSION

Using a conventional monolayer process includes multiple steps of cell expansion, dissociation, phenotype evaluation, banking and finally differentiation towards target functional cells. Reprogramming cells from only a few samples from a single patient requires a full‐time dedicated expert over a costly 2–3‐month period. Recently, scientists have attempted to develop more efficient systems that allow for high‐throughput generation of iPSCs for industrial or clinical use.^30^, ^31^, ^32^ However, these still rely on conventional monolayer reprogramming and are thus relatively slow, inefficient and with high demands for space and manpower. Overcoming these challenges would rapidly push the iPSC field towards safer and more scalable reprogramming methods. To date, only two groups have demonstrated reprogramming performed as cell aggregates in stirred suspension bioreactors. However, these studies were still limited in the sense that they were performed using mouse fibroblasts and cultured as cell aggregates but not demonstrated for human cell lines.^11^, ^12^, ^13^

Here, we have utilized an agitated MC suspension platform, *RepMC*, using 5 sources of human adherent and suspension somatic cells, to enhance the transduction efficiency by about 2‐fold and the reprogramming efficiency by approximately 20‐ to 50‐fold compared to conventional static MNL platforms (*RepMNL*; Table 1). Thus, around 30‐ to 50‐fold more iPSC clones can be generated (Table 1). The resulting MC‐iPSCs possess pluripotency and high differentiation potential (Figures S4 and S5), display a normal karyotype (Figure S6), and show complete absence of Sendai virus in passage 15 cells (Figure S7). Our findings are in agreement with previous reports in which agitation can enhance the transduction and reprogramming efficiency.^33^, ^34^ Hence, we believe that this novel MC reprogramming approach, *RepMC*, has the potential to streamline the iPSC manufacturing process from cellular reprogramming, iPSC expansion, quality assurance, master/working cell banking and directed differentiation to a relevant functional cell type without time‐consuming and laborious processes such as single‐cell dissociation for subculturing followed by re‐aggregation on separate plates as EBs. Importantly, no trypsinization is required in our *RepMC* platform from selection of positive clones to expansion of sufficient cell numbers for characterization and banking. This would also result in an overall less time‐consuming process, especially for cellular differentiation (Table 1). Interestingly, an abnormal trisomy was observed in chromosome 12 in one MNL‐derived clone (Figure S1E), whereas all clones are normal from *RepMC* platform (Figure S6). It may be due to the use of enzymes for cell detachment in monolayer cultures. Enzymatic dissociation used for cell detachment have been reported as one of the major factors affecting the maintenance of genome integrity during culture. Mechanical methods by pipetting are considered as better for preserve genome integrity.^22^, ^35^, ^36^, ^37^ Thus, *RepMC* could potentially offer greater genome stability in addition to the aforementioned benefits. However, more studies are needed to confirm this advantage.

In order to reveal the main impact of agitated MC culture on reprogramming, we compared a set of known genes found in literature commonly associated with the three phases of reprogramming (initiation, maturation and stabilization,^23^, ^24^) between *RepMNL* and *RepMC*. As shown in Figures 3 and 4, early induction of most of the reprogramming‐related genes were observed in *RepMC*, compared to the *RepMNL*. Moreover, in order to further confirm the changes in gene expression over time is not due to the difference in transduction efficiency between methods (Table 1), we performed the same analysis of the expression of reprogramming genes in PBMC (Figure S2), which has similar transduction efficiency between *RepMNL* and *RepMC*. Results obtained are similar to HFF‐01 showing higher expression of various genes in *RepMC*. Particularly, *β‐catenin* was upregulated as early as day 1 (Figure 3). Recent findings demonstrated that shear stress generated by agitation can enhance reprogramming efficiency via mechanosensitive β‐catenin signalling.^38^ Therefore, we hypothesize that agitated MC culture induced early and high expression of *β‐catenin*, which may enhance the expression of pluripotency circuitry genes through interactions with *Klf4*, *Oct4* and *Sox2* to promote cell reprogramming^34^ or enhance Oct‐4 activity and consequently reinforce pluripotency.^39^

Notably, the higher expression of *β‐catenin* could also activate the canonical Wnt signalling pathway.^40^, ^41^ The effects of Wnt/β‐catenin signalling activity on different stages of reprogramming has previously been reported with activation of Wnt signalling during the initiation phase leading to a significant improvement in reprogramming efficiency.^42^ There is evidence to suggest that mechanical stress could induce cellular reprogramming through the Wnt/β‐catenin signalling pathway.^2^, ^38^, ^43^, ^44^ This may also explain the higher reprogramming efficiency without *c‐Myc* in *RepMC* versus *RepMNL* (Table 1) since *c‐Myc* was found to be one of the downstream targets of β‐catenin.^45^ Although *c‐Myc* regulates pathways essential for pluripotency, it is a proto‐oncogene, which hinders clinical applications.^21^ Thus, elimination of *c‐Myc* from the reprogramming system may be important to avoid the tumorigenicity in iPSCs.

An early induction and high levels of E‐cadherin and EpCAM were also observed on day 3, compared to day 7 onwards in *RepMNL* (Figure 4). Teshigawara et al. demonstrated that in human cells, the activation of *E‐cadherin* and *EpCAM*, indicators of the onset of mesenchymal‐to‐epithelial transition (MET), only occurred at the stages of maturation of reprogramming (beyond the first 3 days of the initiation phase), where the cells acquired pluripotency with endogenous Oct4 activation.^24^ The differential timing of entry to MET between the two methods implies that in *RepMC*, cells reached the maturation state earlier during reprogramming shown by the stable expression of *Oct4*, *Sox2* and *Klf4* (Figure 4).

Early induction of *E‐cadherin* and *β‐catenin* expression in *RepMC* (Figure 4) might also enhance the formation of E‐cadherin/catenin complexes resulting in the suppression of epithelial‐to‐mesenchymal transition (EMT), earlier than *RepMNL* (Figure 4). This is followed by remodelling of the cytoskeleton and extracellular matrix (ECM), which have been considered as one of the key rate‐limiting steps in reprogramming.^23^ Early destabilization of the cell's cytoskeleton may favour cytoskeletal reorganization, which could thus facilitate reprogramming.^23^ We hypothesized that reprogramming in agitated suspension culture may allow the cells to change morphology more readily by the rapid induction of cytoskeleton and ECM remodelling. Yet, the precise mechanism requires further investigation. Further experiments should be performed to clarify the basic mechanisms underlying the role of mechanical stimuli in reprogramming processes. In particular, the relationship between E‐cadherin linked to cell–cell interactions and β‐catenin involved in Wnt signalling should be explored due to its likely involvement in enhancing the reprogramming efficiency in *RepMC* culture.

Furthermore, the rapid induction of *Nanog*, *Lin28A* and *Sall4* in *RepMC* culture on day 4 (Figure 4) indicated that the cells entered the maturation phase^23^, ^24^ much earlier than in *RepMNL*, which reached maturation on day 12 or later (Figure 4). Subsequently, the cells enter the stabilization phase, hallmarked by induction of the epigenetic gene *DNMT3B* and accompanied by demethylation of endogenous pluripotency genes *Oct4*, *Sox2* and *Klf4*, on day 7, compared to day 14 onwards in *RepMNL*. Notably, the maturation phase has been identified as the major roadblock for acquisition of pluripotency in cell reprogramming.^46^ We hypothesize that the rapid induction of some of the maturation phase gene markers, *Nanog*, *Lin28A* and *Sall4* improved reprogramming efficiency. Additionally, *Sall4* has also been reported as a reprogramming enhancer. Studies have shown that transduction of *Sall4* gene in mouse and human somatic cells could significantly enhance the efficiency of iPSC generation.^47^, ^48^ We attribute the shear stress generated by agitation as the major trigger for the transcriptional changes observed in the transition from initiation‐to‐maturation phases.

Importantly, *RepMC* can produce iPSCs with high differentiation potential forming all three germ layers and further demonstrated by the formation of functional cardiomyocytes^25^ and erythroblasts.^27^, ^28^ It is worth noting that there is variability between the differentiation efficiencies of different clones (Figure 6). Similar variability was observed using PBMC‐derived hiPSCs generated from monolayer methods, only 1 out of 6 clones have the potential to produce high yields of CD235a+ erythroblasts.^49^ Moreover, a clone that differentiates efficiently to one cell type (e.g., cardiomyocytes) does not necessarily differentiate well to other types (e.g., erythroblasts). Thus, the more cell‐MC aggregates generated, higher the chance to select specific “better” clones that can be differentiated into various lineages.

In conclusion, our study demonstrates that the agitated *RepMC* provides an induction advantage for enhanced iPSC generation. Most importantly, the whole process is cell dissociation free, thus allowing better cell viability and saving in labor and supplies. Moreover, the cell‐MC aggregates can be used for direct differentiation without the need for trypsinization and generation of EBs. Additionally, a larger number of c‐Myc‐free hiPSCs can be generated. Our technology has the potential to accelerate and standardize iPSC research, bringing it to clinical applications more rapidly. However, further experiments should be conducted to evaluate the mechanism of fluid shear‐induced pluripotency and the role of stress responsive genes, focusing on the effects of the Wnt/β‐catenin signalling pathway during reprogramming. Further studies on c‐Myc‐free reprogramming in other cell sources should also be performed. By elucidating the exact mechanism(s) by which liquid shear stress may contribute to promoting pluripotency and preventing differentiation, we will be able to create an efficient environment for both the production of large quantities of pluripotent stem cells, and their differentiated progeny such as heart and blood cells.

## AUTHOR CONTRIBUTIONS

Alan Tin Lun Lam, Shaul Reuveny and Steve Kah Weng Oh were involved in the study design and data analysis. Alan Lam Tin Lun and Valerie Ho performed the experiments. Alan Lam Tin Lun, Shaul Reuveny and Steve Kah Weng Oh wrote the paper with input from Svetlan Vassilev. All authors read and approved the final manuscript.

## CONFLICT OF INTEREST

A patent has been filed on the basis of this work, on which AL‐TL, SR and SK‐WO are named as inventors. SO is co‐founder of Zenzic Labs Pte Ltd and SingCell Tx Pte Ltd.

## Supporting information


**FIGURE S1** Induction of iPSCs from HFF‐01 in *RepMNL*. (A) Representative brightfield images of generating iPSCs in *RepMNL* cultures at different timepoints (days 14, 21, and 28). Images show a representative single colony picked at day 14 (red circle) and plated on a well of LN‐coated plate. Scale bars: 300 μm. (B) Flow cytometry analysis showing expression of pluripotent markers (TRA‐1‐60, Oct4, and SSEA‐4) in the reprogrammed MNL‐iPSCs (MNL01 to MNL04). (C) Log fold‐changes of pluripotent and three germ‐layer‐specific genes compared with undifferentiated MNL‐iPSCs. Spontaneous in vitro differentiation of MNL‐iPSC was carried out with EBs formation. (D) Staining of in vitro differentiated MC‐iPSCs (MNL01) for markers of mesoderm (SMA, α‐smooth muscle actin), ectoderm (β‐III tubulin) and endoderm (AFP, α‐fetoprotein). Scale bars: 200 μm. (E) Karyotyping of MNL01, MNL02, MNL03, and MNL04 clones. MNL02, MNL03, and MNL04 show normal 46 XY karyotypes by G‐banding, 20 metaphase spreads were counted per sample, whereas MNL01 shows an abnormal trisomy in chromosome 12

**FIGURE S2** Gene expression profiles during different phases of PBMC reprogramming determined by qPCR. Log fold‐changes relative to day 0 PBMC are depicted for both *RepMNL* and *RepMC* cultures. Expression levels that differ significantly at matching timepoints were depicted (*T*‐test *p < 0.01; **p < 0.001; ***p < 0.0001). Error bars SD (*n* = 3)

**FIGURE S3** Flow cytometry analysis showing expression of pluripotent markers (TRA‐1‐60, Oct4, and SSEA‐4) in representative reprogrammed MC‐iPSCs from HFF‐01, IMR90, PBMC, CD3+ T cells, and CD34+ cells by the *RepMC*. Bottom right panel shows the representative typical flow cytometric histogram profiles of TRA‐1‐60, Oct4, and SSEA‐4

**FIGURE S4** Characterization of *RepMC* reprogrammed MC‐iPSCs from HFF‐01, IMR90, PBMC, CD3+ T cells, and CD34+ cells MC‐iPSCs by spontaneous in vitro differentiation. The expression of three germ layers markers is analysed by RT‐qPCR. Log fold‐changes of pluripotent and three germ‐layer‐specific genes compared with undifferentiated MC‐iPSCs

**FIGURE S5** Immunostaining of the in vitro differentiated MC‐iPSCs to identify the three germ layers. Mesoderm (SMA, α‐smooth muscle actin), ectoderm (β‐III tubulin) and endoderm (AFP, α‐fetoprotein) were stained. Scale bars: 200 μm

**FIGURE S6** Karyotyping of the representative reprogrammed MC‐iPSCs from HFF‐01, IMR90, PBMC, CD3+ T cells, and CD34+ cells by the *RepMC*. Normal karyotypes by G‐banding, and 20 metaphase spreads were counted per sample

**FIGURE S7** Expression of Sendai virus (SeV) genes in HFF‐01‐derived iPSCs at p3, p6, p10, and p15 for cells reprogrammed in both MNL and MC approaches, measured by RT‐qPCR. SeV gene was retained in both MC‐iPSCs and MNL‐iPSCs up to p10, but not from the cells in passage 15. And there is no difference in the cells generated from both approaches. The Ct (cycle threshold) is defined as the number of cycles required for the fluorescent signal to cross the basal threshold level (default value 0.2)

**TABLE S1** Comparison between non‐agitated and agitated *RepMC* in HFF‐01 fibroblast reprogramming. Transduction efficiency, reprogramming efficiency, and total number of TRA‐1‐60+ clones emerged at day 14 were shown. The reprogramming efficiency was calculated by dividing the number of resulting TRA‐1‐60+ clones at day 14 by the number of input cells and multiplying by 100%. Mean ± SD (*n* = 3)

**TABLE S2** List of primer sets of genes used in this study, which are commonly expressed during the phases of reprogramming

**Appendix S1** Supporting Information

## Data Availability

DATA AVAILABILITY STATEMENT The data used to support the findings of this study are available from the corresponding author upon reasonable request.
