# Supplementary figures and images for: An allied reprogramming, selection, expansion and differentiation platform for creating hiPSC on microcarriers
Source: Cell Prolif. 2022 May 19;55(8):e13256. doi: 10.1111/cpr.13256 (PMC9357361; doi:10.1111/cpr.13256)

## Slide 1
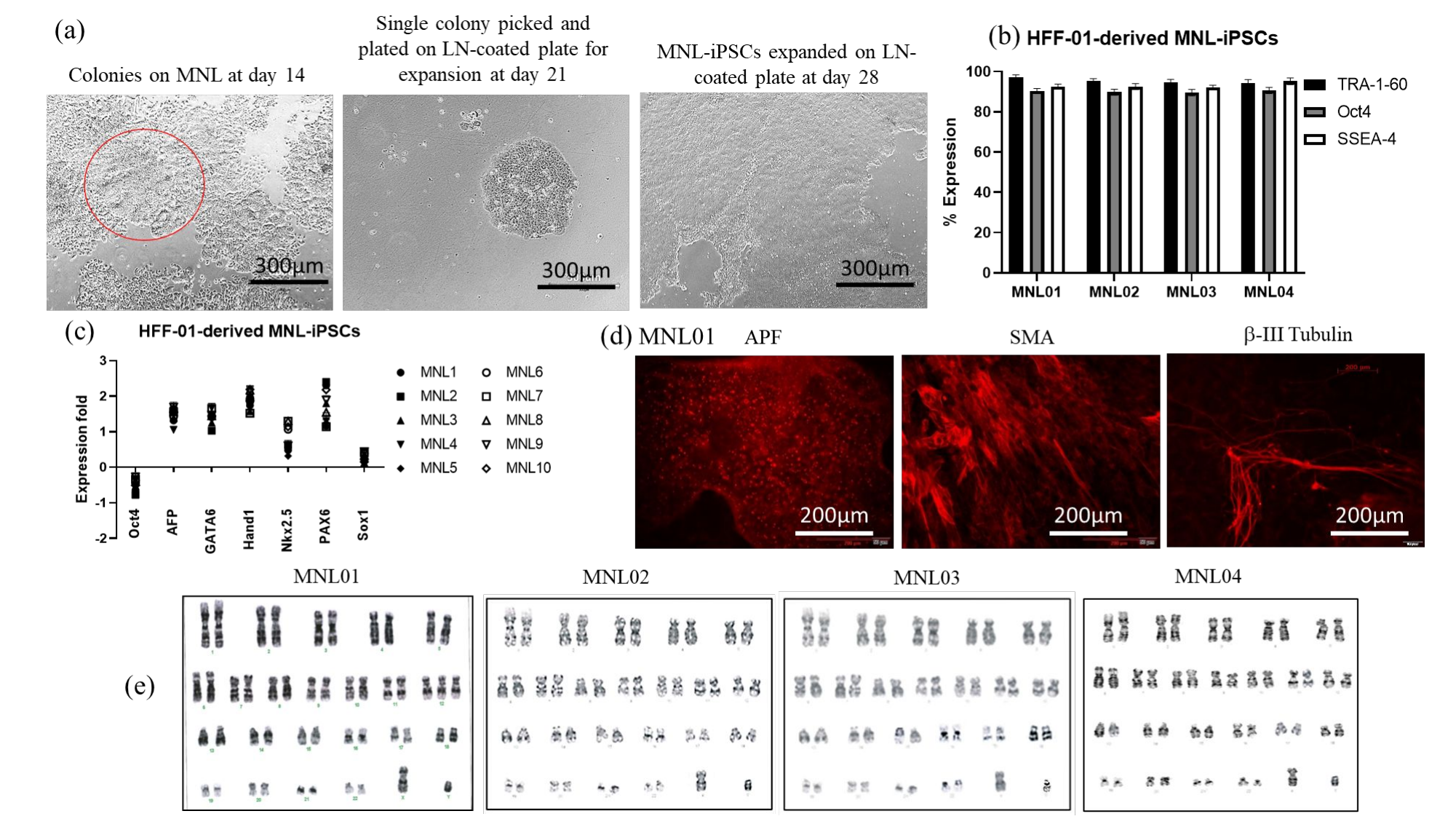

Supplement: Supplementary file 1 — FIGURE S1 Induction of iPSCs from HFF‐01 in RepMNL. (A) Representative brightfield images of generating iPSCs in RepMNL cultures at different timepoints (days 14, 21, and 28). Images show a representative single colony picked at day 14 (red circle) and plated on a well of LN‐coated plate. Scale bars: 300 μm. (B) Flow cytometry analysis showing expression of pluripotent markers (TRA‐1‐60, Oct4, and SSEA‐4) in the reprogrammed MNL‐iPSCs (MNL01 to MNL04). (C) Log fold‐changes of pluripotent and three germ‐layer‐specific genes compared with undifferentiated MNL‐iPSCs. Spontaneous in vitro differentiation of MNL‐iPSC was carried out with EBs formation. (D) Staining of in vitro differentiated MC‐iPSCs (MNL01) for markers of mesoderm (SMA, α‐smooth muscle actin), ectoderm (β‐III tubulin) and endoderm (AFP, α‐fetoprotein). Scale bars: 200 μm. (E) Karyotyping of MNL01, MNL02, MNL03, and MNL04 clones. MNL02, MNL03, and MNL04 show normal 46 XY karyotypes by G‐banding, 20 metaphase spreads were counted per sample, whereas MNL01 shows an abnormal trisomy in chromosome 12 [file CPR-55-e13256-s003.pptx]

## Slide 1
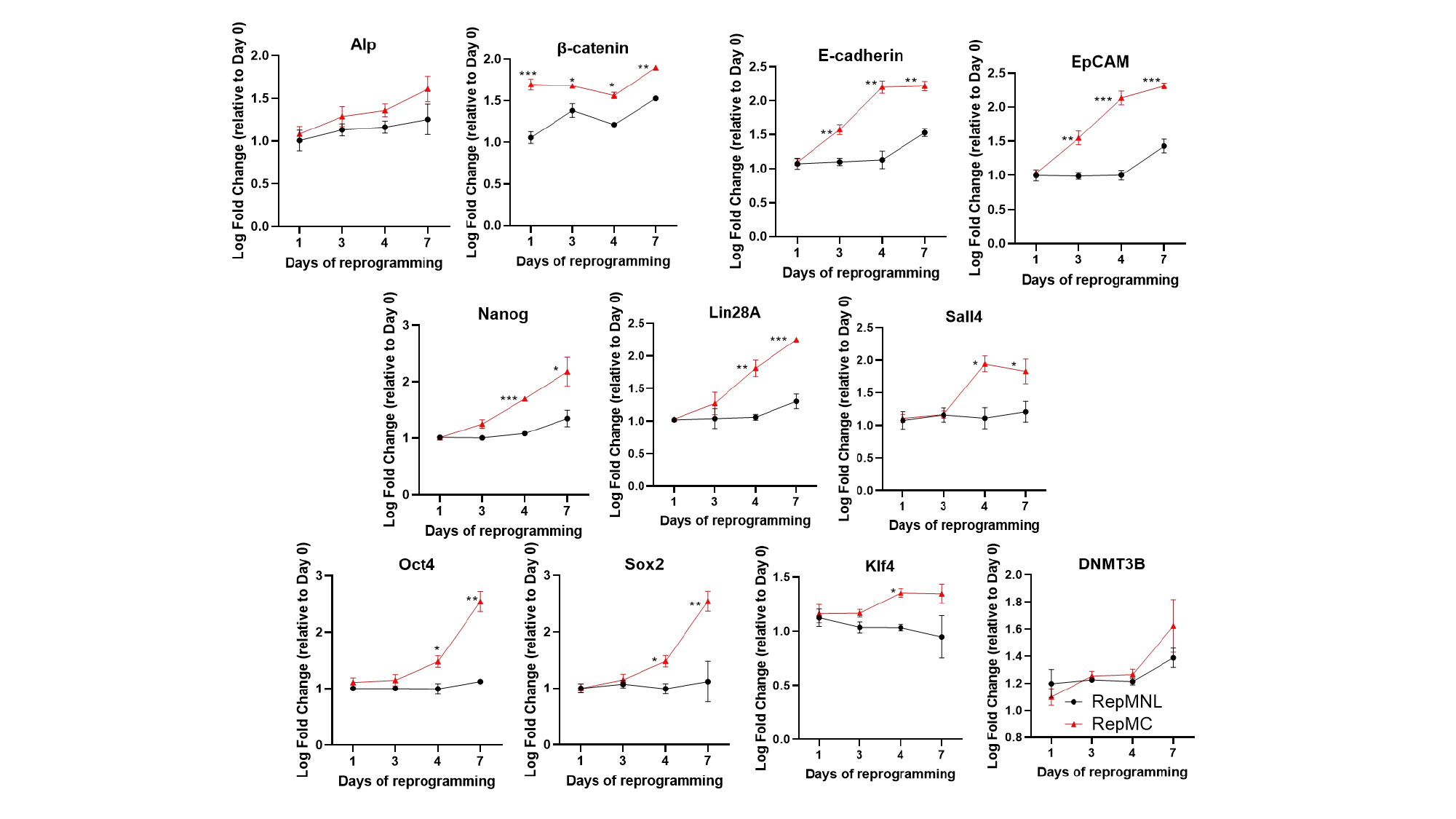

Supplement: Supplementary file 2 — FIGURE S2 Gene expression profiles during different phases of PBMC reprogramming determined by qPCR. Log fold‐changes relative to day 0 PBMC are depicted for both RepMNL and RepMC cultures. Expression levels that differ significantly at matching timepoints were depicted (T‐test *p < 0.01; **p < 0.001; ***p < 0.0001). Error bars SD (n = 3) [file CPR-55-e13256-s006.pptx]

## Slide 1
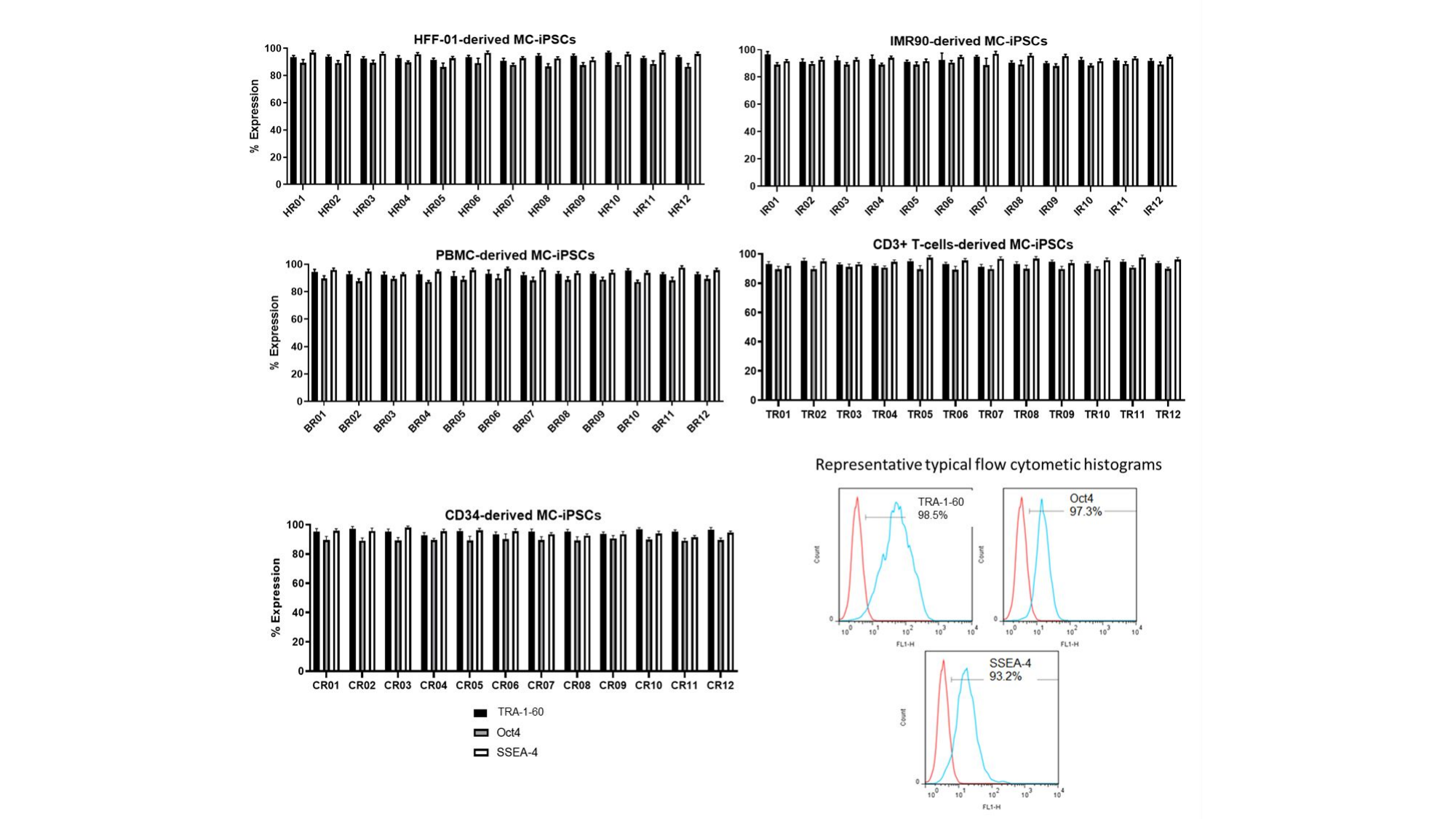

Supplement: Supplementary file 3 — FIGURE S3 Flow cytometry analysis showing expression of pluripotent markers (TRA‐1‐60, Oct4, and SSEA‐4) in representative reprogrammed MC‐iPSCs from HFF‐01, IMR90, PBMC, CD3+ T cells, and CD34+ cells by the RepMC. Bottom right panel shows the representative typical flow cytometric histogram profiles of TRA‐1‐60, Oct4, and SSEA‐4 [file CPR-55-e13256-s010.pptx]

## Slide 1
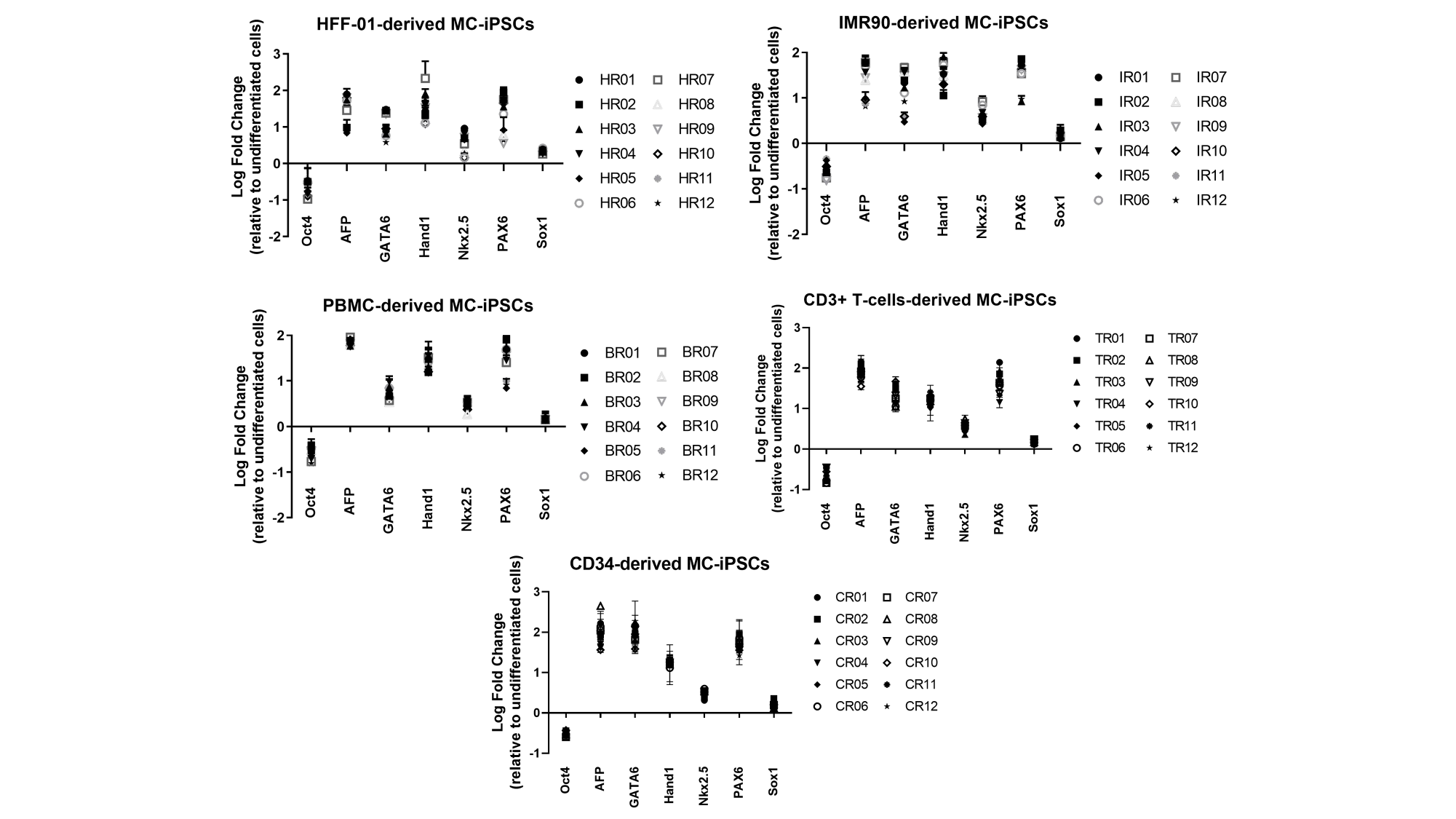

Supplement: Supplementary file 4 — FIGURE S4 Characterization of RepMC reprogrammed MC‐iPSCs from HFF‐01, IMR90, PBMC, CD3+ T cells, and CD34+ cells MC‐iPSCs by spontaneous in vitro differentiation. The expression of three germ layers markers is analysed by RT‐qPCR. Log fold‐changes of pluripotent and three germ‐layer‐specific genes compared with undifferentiated MC‐iPSCs [file CPR-55-e13256-s001.pptx]

## Slide 1
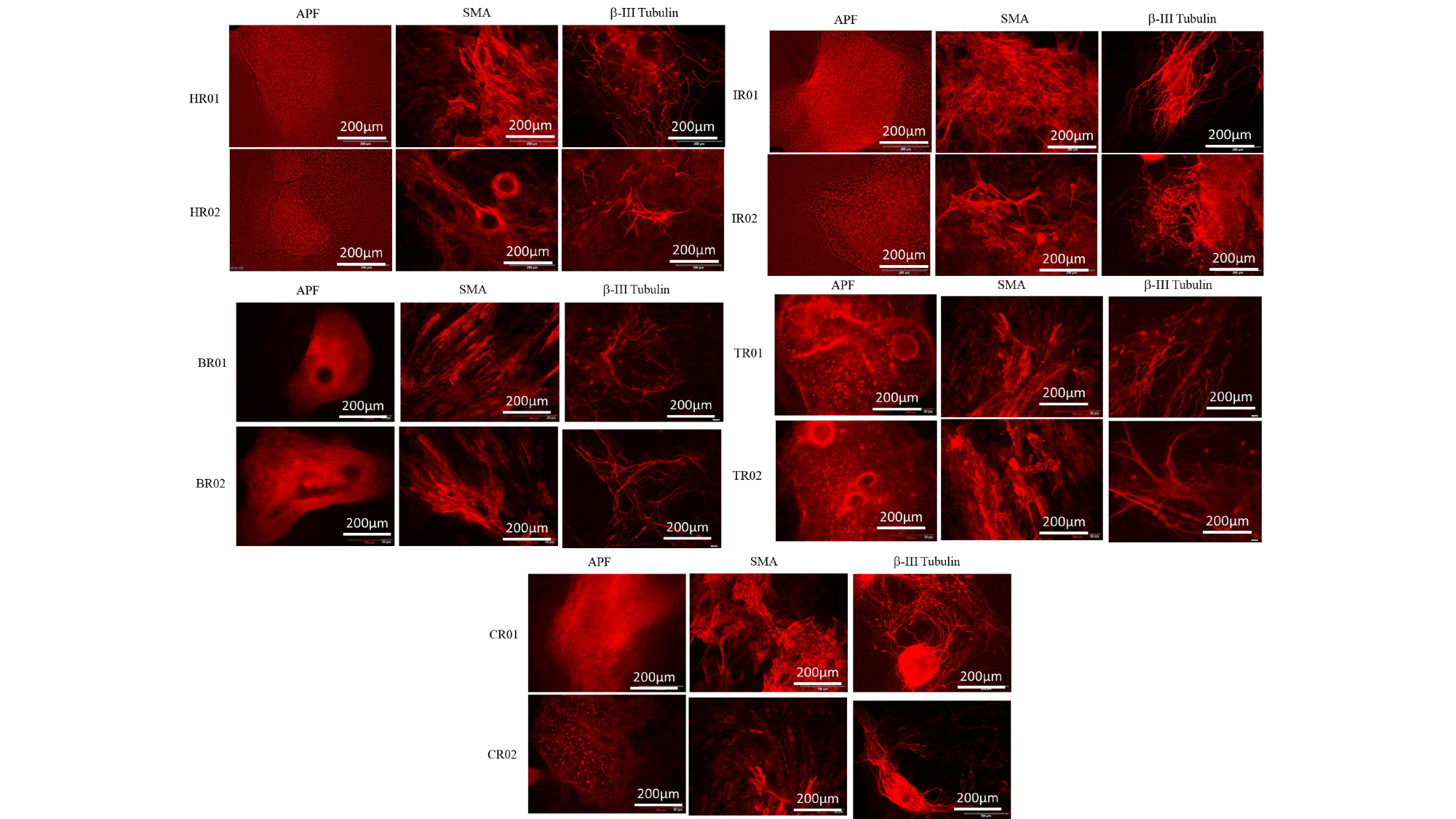

Supplement: Supplementary file 5 — FIGURE S5 Immunostaining of the in vitro differentiated MC‐iPSCs to identify the three germ layers. Mesoderm (SMA, α‐smooth muscle actin), ectoderm (β‐III tubulin) and endoderm (AFP, α‐fetoprotein) were stained. Scale bars: 200 μm [file CPR-55-e13256-s002.pptx]

## Slide 1
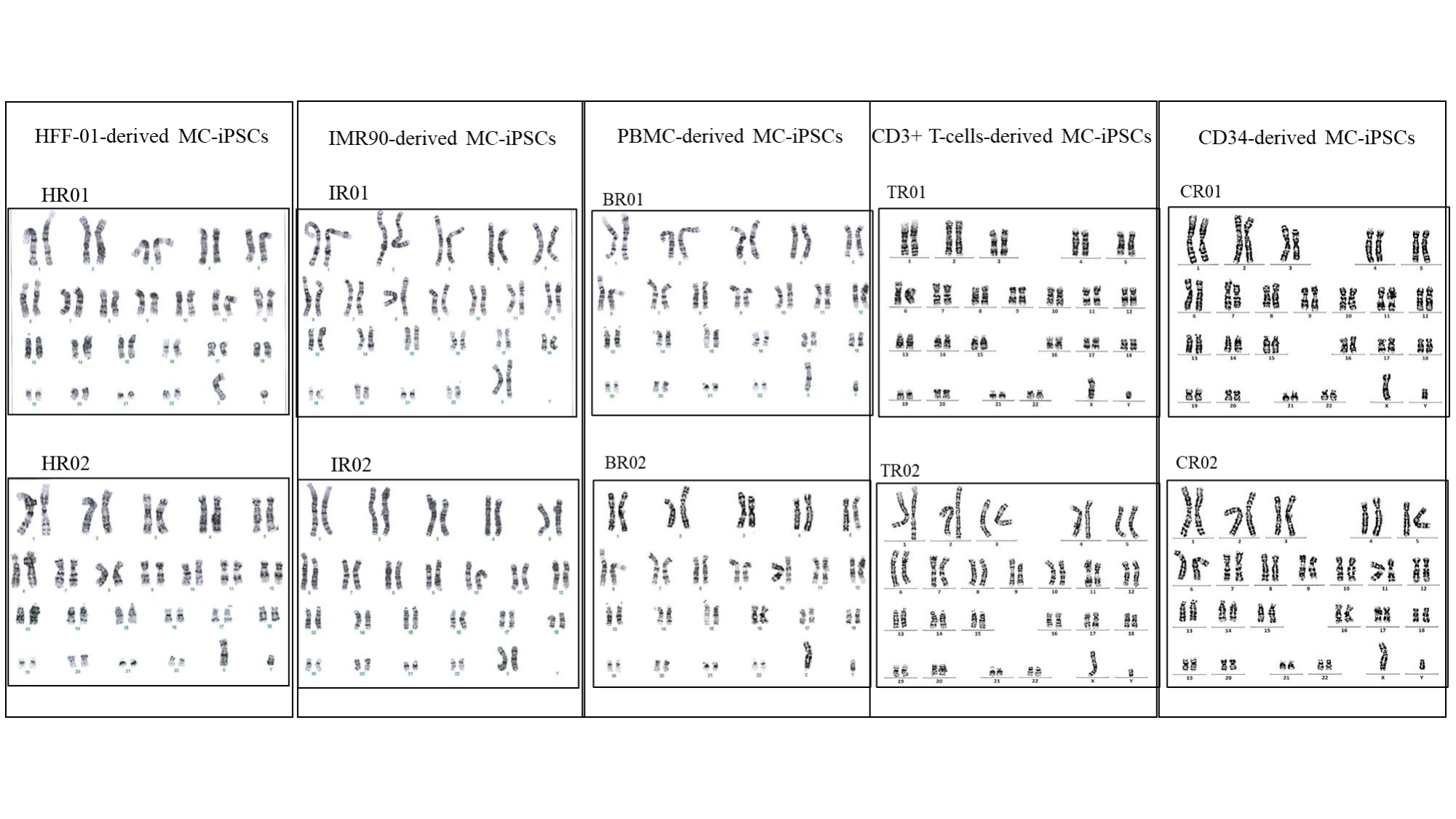

Supplement: Supplementary file 6 — FIGURE S6 Karyotyping of the representative reprogrammed MC‐iPSCs from HFF‐01, IMR90, PBMC, CD3+ T cells, and CD34+ cells by the RepMC. Normal karyotypes by G‐banding, and 20 metaphase spreads were counted per sample [file CPR-55-e13256-s007.pptx]

## Slide 1
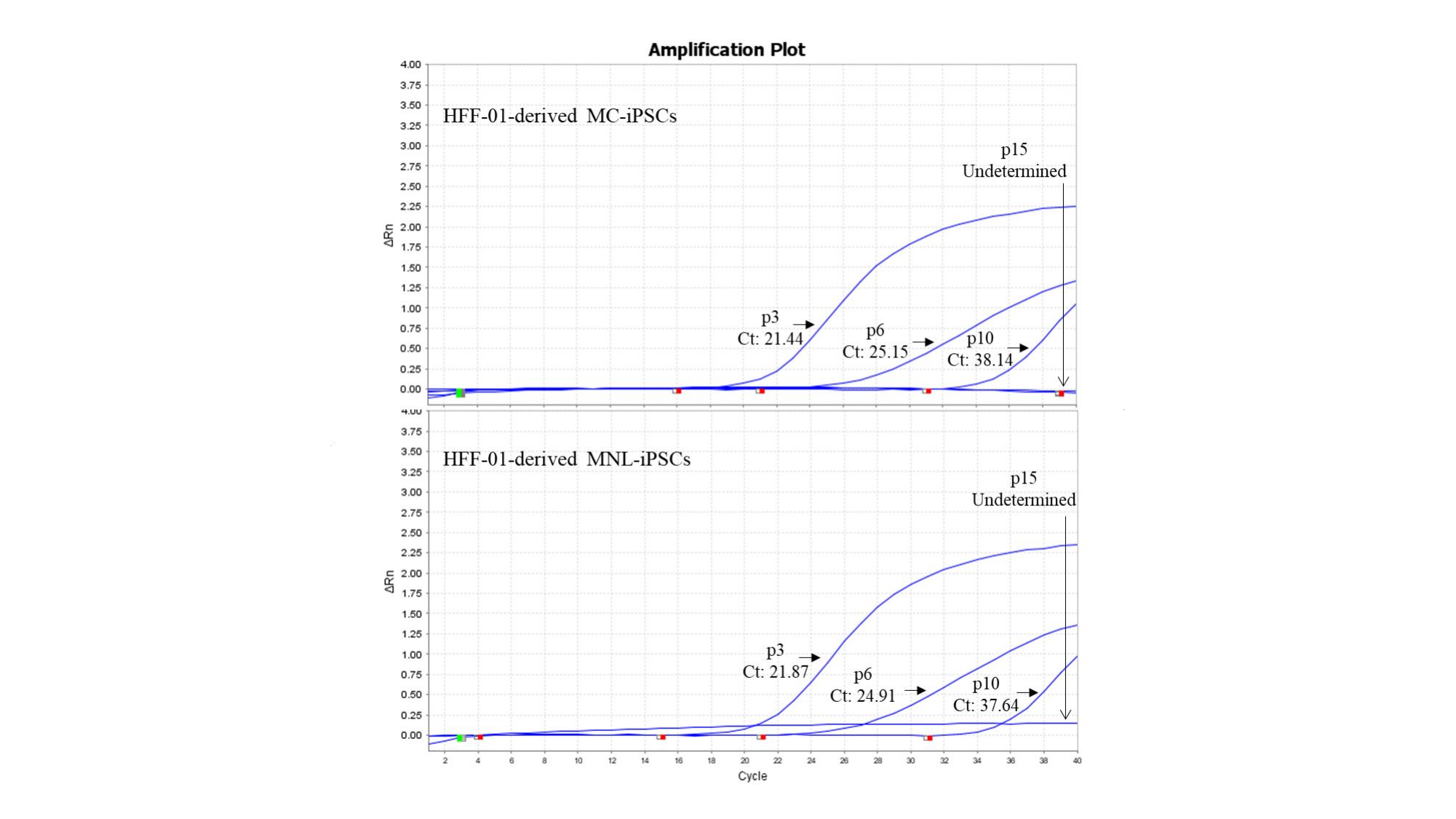

Supplement: Supplementary file 7 — FIGURE S7 Expression of Sendai virus (SeV) genes in HFF‐01‐derived iPSCs at p3, p6, p10, and p15 for cells reprogrammed in both MNL and MC approaches, measured by RT‐qPCR. SeV gene was retained in both MC‐iPSCs and MNL‐iPSCs up to p10, but not from the cells in passage 15. And there is no difference in the cells generated from both approaches. The Ct (cycle threshold) is defined as the number of cycles required for the fluorescent signal to cross the basal threshold level (default value 0.2) [file CPR-55-e13256-s004.pptx]
