# Supplementary material for: An allied reprogramming, selection, expansion and differentiation platform for creating hiPSC on microcarriers
Source: Cell Prolif. 2022 May 19;55(8):e13256. doi: 10.1111/cpr.13256 (PMC9357361; doi:10.1111/cpr.13256)
Supplement: Supplementary file 8 — TABLE S1 Comparison between non‐agitated and agitated RepMC in HFF‐01 fibroblast reprogramming. Transduction efficiency, reprogramming efficiency, and total number of TRA‐1‐60+ clones emerged at day 14 were shown. The reprogramming efficiency was calculated by dividing the number of resulting TRA‐1‐60+ clones at day 14 by the number of input cells and multiplying by 100%. Mean ± SD (n = 3) [file CPR-55-e13256-s009.docx]

Table S1. Comparison between non-agitated and agitated RepMC methods in HFF-01 fibroblast reprogramming. Transduction efficiency, reprogramming efficiency, and total number of TRA-1-60+ cells clones emerged at day 14 were shown. The reprogramming efficiency was calculated by dividing the number of resulting TRA-1-60+ clones at day 14 by the number of input cells and multiplying by 100%. Mean ± SD. (n=3).

|  | Non-agitated RepMC | Agitated RepMC | Fold Change:  Non-agitated *vs* Agitated |
| --- | --- | --- | --- |
| Transduction efficiency (%) | 40.7±2.1 | 60.5±1.8 | 1.5±0.1 |
| Reprogramming efficiency (%) | 0.08±0.01 | 0.90±0.04 | 11.5±1.4 |
| Total no. of TRA-1-60+ cells | 96±9 | 1650±120 | 17.3±1.9 |
