# Supplementary material for: An allied reprogramming, selection, expansion and differentiation platform for creating hiPSC on microcarriers
Source: Cell Prolif. 2022 May 19;55(8):e13256. doi: 10.1111/cpr.13256 (PMC9357361; doi:10.1111/cpr.13256)
Supplement: Supplementary file 9 — TABLE S2 List of primer sets of genes used in this study, which are commonly expressed during the phases of reprogramming [file CPR-55-e13256-s008.docx]

| Gene | Forward primers (5’-3’) | Reverse primers (5’-3’) |
| --- | --- | --- |
| Thy1 | GAAGGTCCTCTACTTATCCGCC | TGATGCCCTCACACTTGACCAG |
| Snail1 | TGCCCTCAAGATGCACATCCGA | GGGACAGGAGAAGGGCTTCTC |
| Snail2 | ATCTGCGGCAAGGCGTTTTCCA | GAGCCCTCAGATTTGACCTGTC |
| CD44 | CCAGAAGGAACAGTGGTTTGGC | ACTGTCCTCTGGGCTTGGTGTT |
| Alp | CCTGATGGAGATGACAGAGGCT | TCAGTGAGTGCCTGGTAAGCCA |
| β-catenin | CACAAGCAGAGTGCTGAAGGTG | GATTCCTGAGAGTCCAAAGACAG |
| Nanog | CTCCAACATCCTGAACCTCAGC | CGTCACACCATTGCTATTCTTCG |
| Lin28A | CCAGTGGATGTCTTTGTGCACC | GTGACACGGATGGATTCCAGAC |
| Sall4 | GAAACCACATCCTTCCAGGCAC | GATAAACGTGGAAGGGAGACTG |
| E-cadherin | CCTCCTGAAAAGAGAGTGGAAG | TGGCAGTGTCTCTCCAAATCCG |
| EpCAM | GCCAGTGTACTTCAGTTGGTGC | CCCTTCAGGTTTTGCTCTTCTCC |
| OCT4 | CCTGAAGCAGAAGAGGATCACC | AAAGCGGCAGATGGTCGTTTGG |
| SOX2 | GCTACAGCATGATGCAGGACCA | TCTGCGAGCTGGTCATGGAGTT |
| Klf4 | CATCTCAAGGCACACCTGCGAA | TCGGTCGCATTTTTGGCACTGG |
| DNMT3B | TAACAACGGCAAAGACCGAGGG | TCCTGCCACAAGACAAACAGCC |
| SeV | GGATCACTAGGTGATATCGAG | ACCAGACAAGAGTTTAAGAGATATGTATC |
| GAPDH | GTCTCCTCTGACTTCAACAGCG | ACCACCCTGTTGCTGTAGCCAA |

Table S2: List of primer sets of genes used in this study, which are commonly expressed during the phases of reprogramming.
