# Supplementary material for: An allied reprogramming, selection, expansion and differentiation platform for creating hiPSC on microcarriers
Source: Cell Prolif. 2022 May 19;55(8):e13256. doi: 10.1111/cpr.13256 (PMC9357361; doi:10.1111/cpr.13256)
Supplement: Supplementary file 10 — Appendix S1 Supporting Information [file CPR-55-e13256-s005.docx]

# Supplemental Information

# An Allied Reprogramming, Selection, Expansion and Differentiation platform for creating hiPSC on Microcarriers

# Alan Lam Tin Lun, Valerie Ho, Svetlan Vassilev, Shaul Reuveny, and Steve Kah Weng Oh

# Supplemental Experimental Procedures

# Cell culture

Human fibroblast lines HFF-1 (ATCC® SCRC-1041™) and IMR-90 (ATCC®CCL-186™) were propagated using α-MEM media containing 10% Fetal Bovine Serum (FBS) and 1% Penicillin-Streptomycin (PS) (designated as α10) in a 37^o^C 5% CO_2_ humidified incubator. Passage 2-5 was used for reprogramming. Single cell suspension was generated by using 0.05% trypsin/0.025% EDTA (ThermoFisher Scientific).

Human frozen PBMCs were purchased from ATCC (PCS-800-011™) and were cultured in PBMC medium consisting of StemPro®-34 serum-free medium supplemented with stem cell factor (SCF, 100 ng/mL), Flt-3 Ligand (Flt-3L; 100 ng/ml), interleukin (IL)-3 (20 ng/ml), and IL-6 (10 ng/ml; all from Peprotech) for 4 days at 37^o^C in 5% CO_2_ incubator for cell recovery. CD3+ T-cells were isolated from the cultured PBMCs by using EasySep Human T-cells isolation kit (StemCell Technologies), according to the manufacturer’s instruction. Isolated CD3+ T-cells were cultured in X-VIVO™ 10 medium (Lonza) supplemented with IL-2 (50 IU per 10^6^ cells; Peprotech) for 4 days at 37^o^C in 5% CO_2_ incubator. CD34+ cells from PBMC were isolated using CD34 MicroBead Kit (Miltenyi Biotech) according to the manufacturer’s protocol. Isolated CD34+ cells were expanded in StemSpanII Serum-Free Expansion Medium (SFEM) (StemCell Technologies), supplemented with Flt3, SCF, TPO (300 ng/ml each), IL-6 (100 ng/ml) and IL-3 (10 ng/ml) (all from Peprotech), for 4 days at 37^o^C in 5% CO_2_ incubator.

**Materials**

CytoTune®-iPS 2.0 Sendai virus Reprogramming kit was purchased from ThermoFisher Scientific. The Sendai virus encodes the four Yamanaka transcription factors: hOct4, hSox2, hKlf4 (hKOS) and hc-Myc. The calculated volumes of each of the three SeV vectors (hKOS, hc-Myc, and hKlf4) were added to the cells using a Multiplicity of Infection (MOI) of 5:5:3, respectively.

Solohill® Plastic Plus (PP+) microcarriers was purchased from Sartorius. Laminin521 (LN) was bought from Biolamina. Chemically synthesized thermoreversible hydrogel (TGP; #5180) was obtained from Mebiol Inc. The gel was prepared according to the manufacturer’s instruction, in brief, the gel was liquefied by adding 10 ml of mT media in the flask to the lyophilized TGP and incubated at 4^o^C overnight for complete dissolution.

**Preparation of LN521-coated MCs and plates**

Prior to the start of the experiment, Solohill® PP+ MCs were prepared according to the manufacturer’s instructions, including sterilization. MCs were coated with LN as previously described.^18^ Briefly, MC coating was prepared by adding 20 µg pf LN521 in 20 mg of Solohill® PP+ in PBS at 4^o^C overnight under rotation. The coated MCs was then designated as LN-coated MCs. Plates were coated at 0.5 µg LN per cm^2^ according to manufacturer’s instructions.

**Sendai virus (SeV) reprogramming**

**Figure. 1** Visual representation of reprogramming methods

***Reprogramming by conventional method in monolayer cultures (****RepMNL****)***

*Adherent HFF-1 and IMR90 fibroblasts:* Transduction was done by adding 4 µg polybrene (Sigma-Aldrich) and SeV vectors encoding the 4 transcription factors (hOct4, hSox2, hKlf4, and hc-Myc) or 3 transcription factors (hOct4, hSox2, and hKlf4) to 3×10^5^ cells and maintained in a well of a 6-well tissue culture plate containing 1 ml of α10 medium. Following overnight incubation (day 1 post-transduction), the spent medium (with SeV) was replaced with fresh α10 medium every other day for a week. Seven days after transduction (day 7), the cells were trypsinized and plated onto a separate well in a 6-well tissue LN-coated plate. On the next day, spent medium was replaced with Essential 8™ medium (E8; ThermoFisher Scientific). Medium was changed daily. Colonies with an iPSC-like appearance were thereafter manually isolated based on morphology and cultured as iPSCs on LN-coated plate with 5ml of mTeSR™1 medium (mT; StemCell Technologies).

*Suspension PBMC, CD3+ T-cells and CD34+ cells:* Suspended 5×10^5^ PBMC, CD3+ T-cells or CD34+ cells were plated per well of a 24-well ULA plate in 500 µl of their corresponding cell growth medium. Subsequently, the SeV vectors encoding 4 transcription factors were added and the plates were placed in a CO_2_ incubator overnight. Following the overnight incubation (day 1 post-transduction), the spent medium (with SeV) was removed by centrifugation and replaced with fresh corresponding cell growth medium. Plates were placed in a CO_2_ incubator for 2 days. Thereafter, the cultures were treated as described above for adherent cells.

***Reprogramming by novel approach in microcarrier cultures (RepMC; Figure 1b)***

Single-cell suspensions of 3×10^5^ HFF-1, IMR90, PBMC, CD3+ T-cells or CD34+ cells were plated per well of a 6-well ULA plate with 2 ml of the corresponding cell growth medium (without MCs). Subsequently, the cell suspension was transduced with SeV vectors encoding hKOS, hc-Myc, and hKlf4 and placed in the CO_2_ shaker incubator under agitation (100-110 rpm) (day 0). After 24 hours (day 1 post-transduction), the SeV-contained spent medium was removed by centrifugation and the culture was replenished with fresh corresponding cell growth medium (2 ml) and transferred to a well of 6-well ULA containing 20 mg of LN-coated MCs and 5 ml E8 medium. The cell-MC were cultured in the CO2 shaker incubator under agitation (100-110 rpm) with fresh E8 medium changed every other day for a week. Seven days after transduction (day 7 post-transduction), the cell-MC were collected, resuspended in 1 ml of mT medium, and subsequently mixed with 3 ml of liquefied TGP hydrogel (1:3) on ice. Immediately, the cell-hydrogel mixture was transferred evenly into 6 wells of a 6-well tissue culture plate. Since the hydrogel is temperature-sensitive, all procedures were done on ice. Thereafter, the plate was put in room temperature for 10 minutes to allow the TGP hydrogel to solidify. mT medium was then overlaid on the TGP hydrogel and the plate was incubated in a 37^o^C, 5% CO2 incubator for 7 days with daily mT medium changes.

***Live-cell immunofluorescence staining of TRA-1-60 positive cells on MC in hydrogel***

Live-staining with StainAlive TRA-1-60 (DyLight™488; Stemgent) was used to identify the onset of TRA-1-60 expression, a marker associated with pluripotency. Briefly, fresh mT media containing 5 µg of StainAlive TRA-1-60 antibody was added to the hydrogel culture and incubated for 30 minutes in a 37^o^C 5% CO_2_ incubator. After two washes with fresh warm mT medium, the TRA-1-60-stained cells in the hydrogel were analyzed using ClonePix™ System (Molecular Devices).

***Selection and expansion of TRA-1-60 positive cell-MC***

Following live stains with StainAlive TRA-1-60 antibody, the positively stained cell-MC (green color) were identified and marked for picking using ClonePix™ System. The marked cell-MC were picked accordingly and transferred into a separate well of a 96-well ULA plate (with 200 µl mT and 0.5 mg LN-coated MCs) using 200 µl pipette tips (tips were changed after each pick to avoid cross-contamination of cells). The 96-well ULA plate was then incubated in a 37^o^C 5% CO_2_ incubator for 7 days under static conditions. On day 7, live-staining with StainAlive TRA-1-60 was performed again in the 96-well ULA plate to identify the growing pluripotent cell on MCs (in aggregates) under a fluorescence microscope. The growing and TRA-1-60+ cell-MC aggregates were selected (size increase at least 2x that of the initial aggregate) and transferred into a separate well of a 12-well ULA plate (with 3 ml mT and 8 mg LN-coated MCs), using 1ml pipette tips to avoid breaking down the cell-MC aggregates. The 12-well ULA plate was then incubated in a 37^o^C 5% CO_2_ incubator for another 7 days under static conditions. After 7-days incubation, fast-growing cell-MC aggregates (size increase at least 2x of initial aggregate plated in the 12-well ULA plate) were selected and transferred into a separate well of 6-well ULA plate (with 5 ml mT and 20 mg LN-coated MCs), using 1 ml pipette tips. The cell aggregates should break down into smaller aggregates gently by the 1 ml pipette tips. The 6-well ULA plate with cell-MC aggregates was then incubated in a 37^o^C 5% CO_2_ incubator for another 7 days under agitation (100-110 rpm). The expanded cell-MC aggregates (MC-iPSCs) were then harvested for characterization.

***Transduction and Reprogramming efficiency***

Transduction efficiency was evaluated by the expression of enhanced green fluorescent protein (GFP) using Hoechst 33342 (ThermoFisher Scientific) and propidium iodide (PI; ThermoFisher Scientific) by an image cytometry, NucleoCounter® NC-3000 (ChemoMetec) according to the manufacturer’s instruction. Briefly, cells were transduced with CytoTune®-EmGFP Sendai Fluorescence Reporter (ThermoFisher Scientific) by MNL and ReprograMC approaches as above mentioned. After 24 hours of transduction, cells were collected and stained with 10 µg/ml Hoechst 33342 for 15 minutes. PI (10 µg/ml) was then added prior to loading into the Nucleocounter. Images acquired from the NucleoCounter were then quantified by the NucleoView™ software. In the software, cells were located using Hoechst 33342 and non-viable cells were stained with PI. The percentage of GFP expressing live cells was then calculated by the NucleoView™ software.

Reprogramming efficiency was calculated as the number of emerging TRA-1-60+ iPSCs colonies (ReprograMNL) or aggregates (ReprograMC) at day 14 per starting cell number (day 0).

Reprogramming efficiency = $\frac{No. of TRA-1-60 iPSCs at day 14}{No. of input starting cells at day 0} x 100\%$

***Time course gene expression analysis***

Gene expression by real-time polymerase chain reaction (RT-qPCR) was measured at multiple time points during cellular reprogramming. A set of known genes related to cellular reprogramming were examined.^23, 24^ Briefly, RNA was extracted from cells at different time points after transduction (days 1, 2, 3, 4, 7, 14, 21, and 28) using an RNA extraction kit (RNeasy Mini Kit; Qiagen) in accordance with the manufacturer’s instructions. The RNA was reverse transcribed into cDNA using Superscript II Reverse Transcriptase (ThermoFisher Scientific). The cDNA was mixed with Power SYBR Green PCR Master Mix (ThermoFisher Scientific) and 200 nM of the specific primers of the genes listed in Supplementary Table S2. The reaction was carried out on an Applied Biosystems™ QuantStudio™ 3 Real-Time PCR System using the following cycling conditions: 50^o^C for 2 minutes, 95^o^C for 10 minutes, following by 40 cycles of 95^o^C for 15 second and 60^o^C for 1 minute. Log fold change of each gene was referenced against the same gene 8 hours post-transduction reprogramming.

***Characterization of Reprogrammed MC-iPSCs***

***Flow cytometry***

Flow cytometry analysis was performed with the extracellular antigen TRA-1-60 (Millipore), intracellular transcription factor Oct4 (R&D Systems) and stage-specific embryonic antigen-4 (SSEA-4; ThermoFisher Scientific). Briefly, MC-iPSCs cells were first trypsinized from MCs with TrypLE^TM^ Express to form a single-cell suspension and then filtered through a 40-µm sieve (BD Biosciences) to remove cell debris and microcarriers. For MNL-iPSCs, a single-cell suspension was obtained from monolayer culture using TrypLE^TM^ Express. Thereafter, all samples were fixed and permeabilized with a Fix and Perm Cell Permeabilization reagent kit (ThermoFisher Scientific) according to the manufacturer’s instructions. During the 15-minute permeabilization step, mouse primary antibodies TRA-1-60 (1:50), Oct-4 (1:20), and SSEA-4 (1:100) were incubated together with the kit’s Reagent B. Cells were subsequently washed with 1% BSA/PBS, followed by 15-minute incubation in the dark with a 1:500 dilution of FITC-conjugated goat anti-mouse antibody (DAKO). Finally, cells were washed and resuspended in 1% BSA/PBS for analysis on a NovoCyte Flow Cytometer (ACEA biosciences). Results were analyzed with FlowJo software (Tree Star), with gating selected at the point of intersection between the marker and isotype control.^18^

***Spontaneous in vitro differentiation***

Spontaneous *in vitro* differentiation was carried out to determine whether MC-iPSC cells cultured on MCs retain their ability to differentiate into the three germ layers. Briefly, MC-iPSC aggregates were cultured as EBs for 7 days in differentiation medium [Knockout™ DMEM (Gibco) with 15% FBS (Gibco)] on non-adherent dishes and subsequently re-plated on 0.1% gelatinized plates for another 14 days.

Immunostaining was carried out to identify the three germ layers, with α-smooth muscle actin, SMA (Sigma), β-III tubulin (Millipore), and α-fetoprotein, AFP (Sigma), as previously described.^18^ Briefly, the differentiated cells were fixed with 4% paraformaldehyde for 15 minutes and blocked for 2 hours in PBS containing 0.1% Triton X-100, 10% goat serum, and 1% BSA. Cells were then probed with primary antibodies SMA (1:400), β-III tubulin (1:1000), and AFP (1:250) for 1 hour and secondary FITC-conjugated antibody for another 2 hours at room temperature in dark. A fluorescent mounting medium with DAPI (Vectashield) was added to cover the cells. Following 1-hour incubation the cells were visualized with Axiovert 200M fluorescence microscope (Carl Zeiss).

The expression of three germ layers markers is analyzed by RT-qPCR, according to Heng et al.^17^ Briefly, RNA was extracted from undifferentiated and differentiated MC-iPSCs. The RNA (1µg) was then reverse transcribed into cDNA. Subsequently the cDNA (100ng) of each sample was mixed with Power SYBR Green PCR Master Mix and 200 nM of the specific primers of the following genes, *OCT-4*, *AFP*, *GATA6*, *Hand1*, *Nkx2.5*, *PAX6*, *SOX1*, and *GAPDH* (housekeeping gene), as previously described.^16^ The qPCR reaction was carried out using the following cycling conditions: 50^o^C for 2 minutes, 95^o^C for 10 minutes, following by 40 cycles of 95^o^C for 15 second and 60^o^C for 1 minute. Log fold change of each gene was referenced against the same gene prior to differentiation of MC-iPSCs.

***Karyotype analysis***

To assess chromosomal stability of the MC-iPSCs clones, karyotyping of all clones by G-banding assay at passage 10 (using bromodeoxyuridine/colcemid) was performed by the cytogenetics lab in Singapore General Hospital.

***RT-qPCR verification of SeV removal***

Total RNA was isolated from the HFF-01-derived MC-iPSCs and MNL-iPSCs at p3, p6, p10, and p15 using RNeasy Mini Kit in accordance with the manufacturer’s instructions, as abovementioned. The RNA (1µg) was then reverse transcribed into cDNA. Subsequently the cDNA (100ng) of each sample was mixed with the Power SYBR Green PCR Master Mix and 200 nM of the specific SeV gene primers listed in Supplementary Table S2. The qPCR reaction was carried out on an Applied Biosystems™ QuantStudio™ 3 Real-Time PCR System using the following cycling conditions: 50^o^C for 2 minutes, 95^o^C for 10 minutes, following by 40 cycles of 95^o^C for 15 second and 50^o^C for 1 minute. GAPDH was used as the internal control.

***Cardiac differentiation***

Cardiac differentiation using a Wnt differentiation protocol was previously described.^25^ Briefly, aggregates (used as EBs) of 1×10^6^ MC-iPSCs on MCs were cultivated in RPMI+B27 medium without insulin (IN^-^) supplemented with 12 µM GSK3β inhibitor CHIR99021 (CHIR) (Selleckchem) and 0.6 mM of L-ascorbic acid 2-phosphate (AA) (Sigma-Aldrich, USA), followed by cultivation in RPMI+B27+IN^-^ supplemented with 2.5μM tankyrase inhibitor IWR-1 (Selleckchem). Beating MC-iPSCs harvested on day 14-15 were trypsinized into single cells and subsequently subjected to flow cytometry analysis for the quantification of the expression of Troponin T (cTnT; Biolegend) as described above.

***Hematopoietic stem cells differentiation***

Hematopoietic stem cells differentiation was done using a BMP4-based protocol.^27^ Briefly, aggregates (used as EBs) of MC-iPSCs (1×10^6^ cells/mL) were cultivated in 5 ml of StemLine® II Hematopoietic Stem Cell Expansion medium (SL2; Sigma-Aldrich) supplemented with BMP4 (R&D system), VEGF (Peprotech), Activin A (StemCell Technologies) and CHIR for 1 day under agitation (75 rpm). On day 2, CHIR was removed by adding fresh SL2 with BMP4, VEGF, Activin A, and β-estradiol. On day 3 of differentiation, a single-cell suspension was harvested from the cell-MC aggregates using 1x TrypLE™ Express as described above. Single cells (2.5×10^5^ cells/ml) were then replated in SL2 supplemented with BMP4, VEGF, bFGF, SCF, IGF2 (StemCell Technologies), TPO (Peprotech), Heparin (Sigma), 3-isobutyl-1-methylxamthine (IBMX; Sigma) and β-estradiol for hematopoietic induction, with medium changes every alternative day. On day 11, cells were harvested and analyzed for the expression of CD45 (a hematopoietic stem cell marker; Biolegend) by flow cytometry.

***Statistical analysis***

Unless otherwise specified, all statistical data is the result of 3 independent experimental repeats, in which numbers were collected independently at the conclusion of the experiment. All data are expressed as the mean ± standard deviation (SD) using the statistical software GraphPad Prism®, version 4.1. Statistical significance was determined from at least 3 independent experiments. Comparisons of two data sets were statistically analyzed with Student's t-test. A p-value is shown when the difference between compared groups is significant, with p<0.05, p< 0.01, p< 0.001, and p<0.0001 considered levels of statistically significant differences.
